# Effect of maternal preconceptional and pregnancy micronutrient interventions on children's DNA methylation: Findings from the EMPHASIS study

**DOI:** 10.1093/ajcn/nqaa193

**Published:** 2020-09-05

**Authors:** Ayden Saffari, Smeeta Shrestha, Prachand Issarapu, Sara Sajjadi, Modupeh Betts, Sirazul Ameen Sahariah, Ashutosh Singh Tomar, Philip James, Akshay Dedaniya, Dilip K Yadav, Kalyanaraman Kumaran, Andrew M Prentice, Karen A Lillycrop, Caroline H D Fall, Giriraj R Chandak, Matt J Silver, the EMPHASIS Study Group, Ayden Saffari, Smeeta Shrestha, Prachand Issarapu, Sara Sajjadi, Modupeh Betts, Sirazul Ameen Sahariah, Ashutosh Singh Tomar, Philip James, Akshay Dedaniya, Dilip K Yadav, Kalyanaraman Kumaran, Andrew M Prentice, Karen A Lillycrop, Caroline H D Fall, Giriraj R Chandak, Matt J Silver, Cyrus Cooper, Chiara Di Gravio, Sarah H Kehoe, Ramesh D Potdar, Chittaranjan S Yajnik, Suraj S Nongmaithem, Harsha Chopra, Meera Gandhi, Lovejeet Kaur, Mohammed Ngum, Momodou K Darboe, Gail R Goldberg, Lena Acolatse, Stephen Owens, Ann Prentice, Kate A Ward, Landing M A Jarjou, Ramatoulie Janha

**Affiliations:** MRC Unit The Gambia at the London School of Hygiene and Tropical Medicine, London, United Kingdom; Genomic Research on Complex Diseases (GRC Group), CSIR–Centre for Cellular and Molecular Biology, Hyderabad, India; School of Basic and Applied Sciences, Dayananda Sagar University, Bangalore, India; Genomic Research on Complex Diseases (GRC Group), CSIR–Centre for Cellular and Molecular Biology, Hyderabad, India; Genomic Research on Complex Diseases (GRC Group), CSIR–Centre for Cellular and Molecular Biology, Hyderabad, India; MRC Unit The Gambia at the London School of Hygiene and Tropical Medicine, Fajara, The Gambia; Centre for the Study of Social Change, Mumbai, India; Genomic Research on Complex Diseases (GRC Group), CSIR–Centre for Cellular and Molecular Biology, Hyderabad, India; MRC Unit The Gambia at the London School of Hygiene and Tropical Medicine, London, United Kingdom; Genomic Research on Complex Diseases (GRC Group), CSIR–Centre for Cellular and Molecular Biology, Hyderabad, India; Genomic Research on Complex Diseases (GRC Group), CSIR–Centre for Cellular and Molecular Biology, Hyderabad, India; Department of Physiology, Boston University, School of Medicine, Boston, MA, USA; MRC Lifecourse Epidemiology Unit, University of Southampton, Southampton, United Kingdom; CSI Holdsworth Memorial Hospital, Mysore, India; MRC Unit The Gambia at the London School of Hygiene and Tropical Medicine, London, United Kingdom; MRC Unit The Gambia at the London School of Hygiene and Tropical Medicine, Fajara, The Gambia; University of Southampton, Southampton, United Kingdom; MRC Lifecourse Epidemiology Unit, University of Southampton, Southampton, United Kingdom; Genomic Research on Complex Diseases (GRC Group), CSIR–Centre for Cellular and Molecular Biology, Hyderabad, India; MRC Unit The Gambia at the London School of Hygiene and Tropical Medicine, London, United Kingdom; MRC Unit The Gambia at the London School of Hygiene and Tropical Medicine, London, UK; Genomic Research on Complex Diseases (GRC Group), CSIR–Centre for Cellular and Molecular Biology, Hyderabad, India, and School of Basic and Applied Sciences, Dayananda Sagar University, Bangalore, India; Genomic Research on Complex Diseases (GRC Group), CSIR–Centre for Cellular and Molecular Biology, Hyderabad, India; Genomic Research on Complex Diseases (GRC Group), CSIR–Centre for Cellular and Molecular Biology, Hyderabad, India; MRC Unit The Gambia at the London School of Hygiene and Tropical Medicine, The Gambia; Centre for the Study of Social Change, Mumbai, India; Genomic Research on Complex Diseases (GRC Group), CSIR–Centre for Cellular and Molecular Biology, Hyderabad, India; MRC Unit The Gambia at the London School of Hygiene and Tropical Medicine, London, UK; Genomic Research on Complex Diseases (GRC Group), CSIR–Centre for Cellular and Molecular Biology, Hyderabad, India; Genomic Research on Complex Diseases (GRC Group), CSIR–Centre for Cellular and Molecular Biology, Hyderabad, India; Program in Molecular Medicine, University of Massachusetts Medical School, Worcester, MA, USA; MRC Lifecourse Epidemiology Unit, University of Southampton, Southampton, UK; CSI Holdsworth Memorial Hospital, Mysore, India; MRC Unit The Gambia at the London School of Hygiene and Tropical Medicine, The Gambia; University of Southampton, Southampton, UK; MRC Lifecourse Epidemiology Unit, University of Southampton, Southampton, UK; CSIR–Centre for Cellular and Molecular Biology, Hyderabad, India; MRC Unit The Gambia at the London School of Hygiene and Tropical Medicine, London, UK; MRC Lifecourse Epidemiology Unit, University of Southampton, Southampton, UK; MRC Lifecourse Epidemiology Unit, University of Southampton, Southampton, UK; MRC Lifecourse Epidemiology Unit, University of Southampton, Southampton, UK; Centre for the Study of Social Change, Mumbai, India; Diabetes Unit, KEM Hospital and Research Centre, Pune, India; Genomic Research on Complex Diseases (GRC Group), CSIR–Centre for Cellular and Molecular Biology, Hyderabad, India; Centre for the Study of Social Change, Mumbai, India; Centre for the Study of Social Change, Mumbai, India; Genomic Research on Complex Diseases (GRC Group), CSIR–Centre for Cellular and Molecular Biology, Hyderabad, India; MRC Unit The Gambia at the London School of Hygiene and Tropical Medicine, The Gambia; MRC Unit The Gambia at the London School of Hygiene and Tropical Medicine, The Gambia; MRC Unit The Gambia at the London School of Hygiene and Tropical Medicine, The Gambia; MRC Nutrition and Bone Health Research Group, University of Cambridge, Cambridge, UK; MRC Unit The Gambia at the London School of Hygiene and Tropical Medicine, The Gambia; Institute of Health and Society, Newcastle University, Newcastle, UK; MRC Unit The Gambia at the London School of Hygiene and Tropical Medicine, The Gambia; MRC Nutrition and Bone Health Research Group, University of Cambridge, Cambridge, UK; MRC Life Course Epidemiology Unit, University of Southampton, Southampton, UK; Nutrition and Bone Health, MRC Human Nutrition Research, University of Cambridge, Cambridge, UK; MRC Lifecourse Epidemiology Unit, University of Southampton, Southampton, UK; MRC Unit The Gambia at the London School of Hygiene and Tropical Medicine, The Gambia; MRC Unit The Gambia at the London School of Hygiene and Tropical Medicine, The Gambia

**Keywords:** DNA methylation, micronutrient intervention, epigenetics, epigenome-wide association study, RCT

## Abstract

**Background:**

Maternal nutrition in pregnancy has been linked to offspring health in early and later life, with changes to DNA methylation (DNAm) proposed as a mediating mechanism.

**Objective:**

We investigated intervention-associated DNAm changes in children whose mothers participated in 2 randomized controlled trials of micronutrient supplementation before and during pregnancy, as part of the EMPHASIS (Epigenetic Mechanisms linking Preconceptional nutrition and Health Assessed in India and sub-Saharan Africa) study (ISRCTN14266771).

**Design:**

We conducted epigenome-wide association studies with blood samples from Indian (*n* = 698) and Gambian (*n* = 293) children using the Illumina EPIC array and a targeted study of selected loci not on the array. The Indian micronutrient intervention was food based, whereas the Gambian intervention was a micronutrient tablet.

**Results:**

We identified 6 differentially methylated CpGs in Gambians [2.5–5.0% reduction in intervention group, all false discovery rate (FDR) <5%], the majority mapping to *ESM1*, which also represented a strong signal in regional analysis. One CpG passed FDR <5% in the Indian cohort, but overall effect sizes were small (<1%) and did not have the characteristics of a robust signature. We also found strong evidence for enrichment of metastable epialleles among subthreshold signals in the Gambian analysis. This supports the notion that multiple methylation loci are influenced by micronutrient supplementation in the early embryo.

**Conclusions:**

Maternal preconceptional and pregnancy micronutrient supplementation may alter DNAm in children measured at 7–9 y. Multiple factors, including differences between the nature of the intervention, participants, and settings, are likely to have contributed to the lack of replication in the Indian cohort. Potential links to phenotypic outcomes will be explored in the next stage of the EMPHASIS study.

## Introduction

Maternal nutrition around the time of conception and throughout pregnancy can influence offspring health across the life course (1, 2). A micronutrient-deficient maternal diet has been associated with increased risk of birth defects (3), childhood growth stunting (4), obesity (5), impaired cognitive development (6), and susceptibility to diabetes (7) in later life. In low- and middle-income countries, many women of reproductive age are deficient in a number of key micronutrients (2). Early nutritional intervention may therefore provide an effective means for improving maternal and child health as well as reducing the future burden of noncommunicable diseases (2).

Epigenetic modification has emerged as a plausible mechanism through which early environmental factors, including nutrition, could induce persistent changes in gene expression patterns leading to physiologic changes (8). In the context of nutrition, DNA methylation (DNAm) has received particular attention (9). First, DNAm plays a crucial role in the establishment and maintenance of cell, tissue, and parent-of-origin specific gene expression during embryonic and fetal development (10). Second, evidence from animal and human studies suggests that DNAm is sensitive to early environmental factors, including maternal diet (11). In rodent models, protein restriction during pregnancy is linked to altered DNAm in the offspring, and induced DNAm modifications are associated with physiologic changes, including increased blood pressure and impaired lipid and carbohydrate metabolism that are reversed by supplementation with glycine or folate (12, 13), suggesting an important role for 1-carbon (1-C) metabolites. In humans, observational studies have examined the effect of maternal factors such as low maternal prepregnancy BMI and maternal diet on DNAm in offspring. Maternal micronutrient status has been linked to methylation differences at a number of genes, including *H19*/*IGF2* (14, 15) and *MEG3* (14), which are associated with outcomes including birth weight (14–16). Maternal 1-C metabolites in plasma measured at conception have been associated with methylation differences at several genes (17, 18), including *POMC*, which has been linked to obesity in children and adults (19).

Periconception, spanning the weeks before and after conception, represents a sensitive window in which differences in the parental nutritional milieu have the potential to induce persistent gene regulatory changes in the offspring, with implications for organ development and later health (1). The 1-C metabolic pathway links maternal (and paternal) micronutrient status with the gametic and embryonic milieu and the developing embryonic epigenome (20). Micronutrients play an essential role in the 1-C pathways involved in DNAm, either providing methyl groups (e.g., folate, betaine, methionine, serine) or acting as cofactors (e.g., vitamins B-2, B-6, and B-12) (9). Taken together, the evidence suggests that alterations to 1-C pathways present a plausible link between early maternal nutrition and offspring health via DNAm changes.

Opportunities to study links between early nutrition and DNAm in countries with a high burden of disease and in the context of a randomized preconceptional nutritional intervention are extremely rare. The few randomized controlled trials (RCTs) that have been conducted [see, e.g., (21–23)] have not involved supplementation in the pre/periconceptional period and have been carried out in high-income countries where health outcomes and nutritional intakes are very different from those experienced in low- and middle-income countries.

The EMPHASIS study (Epigenetic Mechanisms linking Preconceptional nutrition and Health Assessed in India and sub-Saharan Africa; ISRCTN14266771) was established to examine the effects of maternal pre- and periconceptional nutrition on genome-wide DNAm in children in an RCT setting and to relate nutrition-related DNAm changes to a range of health outcomes (24). The original intervention trials, one in India and one in sub-Saharan West Africa, were planned independently, and neither were designed with a follow-up epigenetic study in mind. Each involved a different nutritional intervention (food-based in the Indian cohort; micronutrient pill-based in The Gambia) and differed in other respects, including age and nutritional and genetic backgrounds. The study therefore presents an opportunity to compare and contrast the effect of different interventions on diverse cohorts that nonetheless endure a relatively high burden of disease. In the first stage of the study presented here, we report results from our investigation of the influence of maternal nutritional intervention on DNAm in offspring. We conducted an epigenome-wide association study (EWAS) using the Infinium MethylationEPIC array, as well as a candidate gene study at selected loci not present on the EPIC array and previously associated with early nutritional exposures and/or later outcomes of interest (24). The second stage of the study, which is currently under way, will consider links between DNAm and various phenotypic outcomes in the children (24).

## Methods

### Study cohorts and design

The EMPHASIS study follows up 2 cohorts of children born to mothers who took part in separate RCTs of nutritional supplementation before and during pregnancy (24). The original trials were 1) the Mumbai Maternal Nutrition Project (MMNP) (also known as project SARAS; ISRCTN62811278) among women living in slums in the city of Mumbai, India (25), and 2) the Periconceptional Multiple Micronutrient Supplementation Trial (PMMST; ISRCTN13687662) among women living in rural West Kiang, The Gambia (26).

In the Mumbai trial, the intervention was a daily snack, eaten in addition to normal diet, made from naturally micronutrient-rich local foods. The aim was to increase women's frequency of intake of particular foods (green leafy vegetables, fruit, and milk) rather than a specific amount of nutrients, motivated by an earlier Indian observational study that showed that maternal intakes of these foods during pregnancy were low and directly related to newborn size (27). Women were randomized to receive either 1 intervention or 1 control snack daily, 6 d per week, and intake was observed and recorded. Control snacks contained foods of low micronutrient content (e.g., potato, onion). Laboratory analysis (carried out on 5–6 snacks ∼6 monthly during the trial by Eclipse Scientific Group) showed that intervention snacks contained on average 10–23% of the WHO Reference Nutrient Intake (RNI) for β-carotene, vitamins B-2 and B-12, folate, calcium, and iron; 0.7 MJ of energy; and 6 g of protein compared with 0–7% RNI for the micronutrients, 0.4 MJ of energy, and 2 g of protein in control snacks (25). Nonpregnant women were recruited (*N* = 6513) to ensure supplementation during the periconceptional period. Women who became pregnant continued to be offered supplementation until delivery and were also supplied with routine iron (100 mg) and folic acid (500 µg) supplements once pregnancy was diagnosed.

The “dose” of the food supplement in the Mumbai trial was designed to increase women's intake of these foods by 1 portion every alternate day, over and above their habitual diet. Full compliance was therefore defined as consuming at least 3 snacks per week during the periconceptional period (from 3 mo before to 2 wk after conception). Only 43% in the intervention group and 58% in the control group achieved this, whereas approximately two-thirds of women had at least 2 per week and three-quarters had 1 per week (25). There were 1962 singleton live births between 2006 and 2012, of whom 1526 were born to mothers who started supplementation at least 3 mo prior to conception. Supplementation stopped on delivery of the child. The median duration of supplementation up to the last menstrual period in the Mumbai study was 46.1 wk (IQR: 25.9, 85.0 wk). Despite low compliance, the intervention increased birth weight among women who started supplementation at least 3 mo prior to conception; there was also an interaction between intervention group and maternal prepregnant BMI such that the effect of supplementation on birth weight was greater among women of higher BMI (25). The intervention also reduced the incidence of gestational diabetes (28).

In contrast to the Indian study, the intervention in the Gambian trial was designed to provide 1 WHO RNI of the full range of known vitamins and important minerals (in other words 100% RNI), a more commonly used approach and a dose considered safe in pregnancy. In this trial, the intervention was a daily multiple micronutrient tablet [United Nations International Multiple Micronutrient Preparation (UNIMMAP)] providing 1 RNI of vitamin A, vitamin B-1, vitamin B-2, niacin, vitamin B-6, folic acid, vitamin B-12, vitamin C, vitamin D, vitamin E, iron, zinc, copper, selenium, and iodine. Women randomly allocated to the control group received placebo tablets. Nonpregnant women were recruited (*N* = 1156), as in Mumbai. Compliance with supplementation was assessed by fortnightly tablet counts (ratio of number of tablets apparently consumed to number of days enrolled in study) and was estimated to be 88% in the UNIMMAP arm and 86% in the placebo arm. Women stopped the supplement when they became pregnant, confirmed by pregnancy test or ultrasound (approximately 12 wk of gestation). The median time on supplementation before positive pregnancy test was 24.1 wk (IQR: 13.1, 43.1 wk), after which the intervention was stopped and replaced with routine iron (60 mg) and folic acid (250 µg) supplements and antimalarial prophylaxis. There were 376 singleton live births between 2007 and 2008. The intervention reduced uterine artery resistance index between 18 and 32 wk of gestation; it had no effect on birth weight (26).

For this analysis, the Mumbai children have been followed up at 5–7 y of age (“SARAS KIDS” study), and data and samples for the first 700 children studied who were born to women who started supplementation at least 3 mo prior to conception have been used in EMPHASIS. The Gambian children were followed up aged 7–9 y in 2016; all 299 Gambian children retraced from the PMMST group were selected for this first stage of the EMPHASIS study. Cohort characteristics are summarized in **Table 1**. CONSORT flowcharts for each cohort are presented in **Supplemental Figure 1**. Further details on maternal and child characteristics of EMPHASIS participants are included in the EMPHASIS protocol study (24).

**TABLE 1:** Cohort characteristics^1^

	Cohort			
Characteristic	India—MMNP		The Gambia—PMMST	
Type of intervention	Food-based multiple micronutrients		UNIMMAP tablet	
Period of intervention^2^	Preconception >3 mo to birth		Preconception to positive pregnancy test	
EPIC and GSA samples, *n*	698 (327/371)^3^		293 (140/153)^3^	
Technical validation source	Blood (92 samples)		Blood (92 samples)	
Cross-tissue source	NA		Buccal (94 samples)	
Baseline characteristics (post-QC)	Intervention	Control	Intervention	Control
Child age, y	5.76 (5.64, 5.98)	5.74 (5.61, 5.97)	9.01 (8.62, 9.21)	8.98 (8.62, 9.22)
Child sex, male/female, *n*	168/153	209/156	64/74	93/58
Maternal age, y	24.0 (22.0, 24.6)	25.0 (22.0, 28.0)	28.6 (24.9, 34.4)	30.0 (25.5, 33.3)
Maternal height, cm	151.4 (147.5, 155.0)	151.0 (147.5, 155.0)	161.6 (157.6, 165.0)	160.4 (156.7, 163.9)
Maternal BMI, kg/m^2^	19.1 (17.5, 21.8)	19.9 (17.9, 22.8)	20.6 (19.3, 22.5)	20.9 (19.5, 23.4)

^1^Values are presented as median (IQR) unless otherwise indicated. EPIC, Illumina MethylationEPIC BeadChip; GSA, Global Screening Array-24 v1.0 Beadchip (genotyping); MMNP, Mumbai Maternal Nutrition Project, India; NA, nonapplicable; PMMST, Periconceptional Multiple Micronutrient Supplementation Trial, The Gambia; QC, quality control; UNIMMAP, United Nations Multiple Micronutrient Preparation (UNIMMAP) tablet.

^2^Duration of supplementation [median (IQR)]: India 46.1 (25.9, 85.0) wk supplementation start to conception; Gambia: 24.1 (IQR: 13.1, 43.1) wk supplementation start to date of last menstrual period.

^3^Numbers pre-QC (intervention/control).

### Ethics and consent

MMNP (ISRCTN62811278) was approved by the ethics committees of BYL Nair and TN Medical College, Grant Medical College, and Sir JJ Group of Hospitals, Mumbai. PMMST (ISRCTN13687662) was approved by the joint Gambia Government/Medical Research Council (MRC) Unit The Gambia's Ethics Committee. Ethics approval for the follow-up of the children in Mumbai (“SARAS KIDS”) was obtained from the Intersystem Biomedica Ethics Committee, Mumbai on 31 May 2013 (serial no. ISBEC/NR-54/KM/JVJ/2013). Ethics approval for the EMPHASIS study in The Gambia was obtained from the joint Gambia Government/MRC Unit The Gambia's Ethics Committee on 19 October 2015 (serial no. SCC 1441). The EMPHASIS study is registered as ISRCTN14266771. Signed informed consent was obtained from parents and verbal assent from the children.

### Sample preparation, methylation profiling, and quality control

#### Sample preparation

Peripheral blood samples from the children were collected and stored in EDTA-containing vacutainer tubes at −80°C until required. DNA was extracted using the QIAamp Midi DNA isolation kit (Qiagen) according to the manufacturer's protocol. DNA quality and quantity were assessed using Invitrogen Quant-iT PicoGreen (Invitrogen) and Nanodrop 1000 Spectrophotometer (Thermo Fisher Scientific). Samples with low-quality and/or low-quantity DNA were excluded. Buccal DNA extraction was also performed using the QIAamp DNA mini kit.

#### Epigenome-wide DNA methylation profiling

Epigenome-wide DNAm profiling was performed for a total of 698 Indian and 293 Gambian samples using the Illumina MethylationEPIC BeadChip platform (Illumina). The EPIC array covers >850,000 CpGs genome-wide in a variety of contexts, including gene promoters, intra- and intergenic regions, enhancer regions, and transcription factor binding sites. Bead chips were processed following the manufacturer's guidelines. To reduce the potential for confounding by batch effects, samples were distributed on the plates following a balanced, randomized design, specified in advance. Further details on the laboratory protocol are given in the **Supplemental Methods** SM1.1, SM2.1, and SM2.2. The EPIC methylation arrays, pyrosequencing assays, and global screening array (GSA) genotyping arrays were processed at the CSIR–Centre for Cellular and Molecular Biology in Hyderabad, India.

For quality control (QC) and preprocessing, a first-pass inspection of the data was carried out using Illumina's GenomeStudio software. The data generated were found to be of a high quality. The raw intensity .idat data files were then imported into the R statistical environment, and the package *meffil* (29) from *Bioconductor* (30) was used for further preprocessing following the standard *meffil* pipeline. The following sample-based checks were performed (number of samples excluded using *meffil* default cutoffs in parentheses): identification of sex mismatches (5 Indian, 0 Gambian samples) and outlying arrays based on array-wide methylation signal (7 Indian, 4 Gambian samples). Probe-based QC and filtering were also performed to remove probes with (number using *meffil* defaults in parentheses) low detection p or bead numbers (1494 and 2635 probes in Indian and Gambian data, respectively) and mapping to sex chromosomes and/or previously identified as ambiguously mapping (31) (61,523 and 61,225 probes in Indian and Gambian data, respectively). Following these steps, the final dimensions of the data sets were as follows: Indian cohort had 686 samples with measurements for 803,120 probes, and the Gambian cohort had 289 samples with measurements for 802,283 probes.

Next, normalization to reduce technical variability was performed following the standard *meffil* approach. More details on QC and data preprocessing steps are given in the Supplemental Methods SM2.3 and SM2.4.

#### Pyrosequencing of candidate loci

We selected 13 additional loci of interest (from 11 genes) not present on the Illumina EPIC array for pyrosequencing. Loci were prioritized based on the timing and type of in utero exposure, phenotypic effects, and functional links reported in the original studies (**Supplemental Table 1**) (24). Three candidate loci were excluded due to 1) close proximity of target CpGs to EPIC array CpGs (*PPARGC1A*) and 2) technical issues with pyrosequencing (*VIPR2, POMC*). Consequently, a total of 698 samples from India and 292 Gambian samples were assayed for the 10 candidate loci within 8 genes. The list of 10 candidate loci along with the sequencing primers used is given in **Supplemental Table 2**. Pyrosequencing was performed as described for technical validation (Supplemental Methods SM2.5).

##### QC and preprocessing

Methylation values for the loci were exported from the Pyromark q96 SW2.0 software. Assay precision was evaluated by calculating the CV from methylation values of the positive control for each sequenced locus. The CV ranged from 2–5% for most assays (see **Supplemental Table 3**). Density plots were generated to inspect the distribution of methylation values for all pyrosequenced CpGs. Methylation values greater than 3 IQR for a CpG were excluded from downstream analysis if they were also flagged by the Pyromark software as possibly subject to technical errors.

#### Buccal samples for measurement of cross-tissue methylation correlation

Mawi iSwab kits were used for collecting buccal swabs from all children in The Gambia. DNA from buccal swab samples was isolated using the QIAmp DNA mini kit (Qiagen) as per the manufacturer's instructions. A total of 300 ng DNA was bisulfite converted using the EZ-96 DNA Methylation-Goldkit (Zymo Research), and 4 CpGs on *ESM1* and 1 on *LZTS1* were pyrosequenced in 92 samples to assess cross-tissue methylation correlation.

#### Genotyping

Genome-wide single-nucleotide polymorphism (SNP) genotypes for 698 Indian and 293 Gambian samples were generated using the Infinium Global Screening Array-24 v1.0 Beadchip (GSA) array (Illumina). The GSA provides coverage of ∼650,000 SNPs genome-wide combining multiethnic content with curated clinical variants with established disease associations. The arrays were processed following the manufacturer's guidelines (Supplemental Methods SM3).

Initial processing of the GSA array data was performed using the GenomeStudio genotyping module (Illumina). Following published guidelines (32), a total of 5 samples and 38,723 probes were removed from the Indian cohort and 3 samples and 73,873 SNPs were excluded from the Gambian cohort. Prior to methylation quantitative trait loci (mQTL) analysis for intervention-associated loci, the Gambian genotype data underwent further processing using PLINK (33) version 1.9, and SNPs were filtered based on Hardy-Weinberg equilibrium (HWE) *P* < 0.001, minor allele frequency (MAF) <5%, and genotype missingness >5%. A total of 282,399 SNPs were excluded, giving a final total of 290 samples and 286,552 SNPs for the Gambian data set. Last, the processed genotypes were recoded as additive allelic dosages for import into R for subsequent mQTL analysis.

We followed up our discovery of an mQTL proximal to *ESM1* on chromosome 5 in the Gambian data set by performing a high-resolution mQTL scan using imputed genotypes from this chromosome. Chromosome 5 was prephased using SHAPEIT version 2, and imputation was performed using IMPUTE version 2.3.2 on 1000 genome phase 3 data (34). Imputed genotypes for chromosome 5 SNPs with a minimum information score of 0.9 were taken forward for mQTL analysis (731,027 variants for 288 individuals). Further processing was performed using PLINK. The same filters were applied: HWE *P* < 0.001 (379 SNPs removed), MAF <5% (324,709), and genotype missingness >5% (0), leaving a total of 405,939 probes.

### Main statistical analysis and validation

#### Epigenome-wide association studies

##### Site-level differential methylation analysis

A linear regression-based approach was used to identify differentially methylated positions (DMPs) (individual CpGs) associated with the intervention while adjusting for other technical and biological variables. To account for multiple testing, a Benjamini-Hochberg false discovery rate (FDR) threshold of 5% was used. Each cohort was analyzed separately and then the results were compared. See Supplemental Methods SM4.1 and SM4.2 for more detailed descriptions for each of the analyses outlined below. A detailed analysis plan published prior to commencement of all analyses is also available on the EMPHASIS website (www.emphasisstudy.org).

The *limma* package (35) was used to perform linear regressions using a variety of models. Adjustments for batch/technical covariates, cell composition, and other factors used combinations of known covariates and/or derived components/surrogate variables obtained using data reduction/latent variable methods. Model selection was performed using a number of metrics, with post hoc sensitivity analysis to check the robustness of results to model choice (see Supplemental Methods SM4.1 for further details).

##### Region-level differential methylation analysis

The methylation status of adjacent CpG sites can be highly correlated, particularly in the context of larger-scale regulatory features—for example, CpG islands in gene promoters. This property can be used to combine signals from individual sites and provide a more robust and potentially more functionally relevant signal. Because methods for identifying differentially methylated regions (DMRs) rest on different underlying assumptions on what constitutes a regional signal, we used 2 common methods, *DMRcate* (36) and *comb-p* (37), and checked for agreement between both on the grounds that significant findings from both would increase confidence that they were true DMRs (Supplemental Methods SM4.3).

##### Technical validation

Technical validation of identified intervention-associated loci from CpG and region-level analyses was performed by pyrosequencing a subsample of 92 individuals from each cohort and checking correlation between the methylation values obtained on both platforms. Assays were designed and sequencing performed using the PyroMark Q96 MD pyrosequencer (Qiagen) as per the manufacturer's instructions (see Supplemental Methods SM2.5 for details on sample selection, assay design, and validation strategy).

#### Analysis of candidate loci

Individual sites within each candidate region were tested for association with intervention status using robust regressions (R *rlm* function) with methylation as outcome and pyrosequencing batch (plate), age, sex, blood cell counts, and season of conception (Gambia only) as covariates. To obtain a single, regional measure of significance for each candidate gene, statistics from the single-site robust regression analyses were combined using empirical Brown's method (38). This takes into account correlation between measures (methylation values of the CpGs) in the calculation of a combined *P* value. A 5% FDR threshold was used to control for multiple testing across the candidate regions (Supplemental Methods SM5.1 and SM5.2).

### Additional statistical analysis

#### Enrichment tests

The EWAS results lists were tested for enrichment of loci previously associated with periconceptional maternal nutrition to help identify potential subthreshold signals. Here the alternative hypothesis is that a set of loci is jointly more significantly associated with the intervention than would be expected by chance. DMPs at nominal *P* < 0.05 and showing an effect size (Beta difference between intervention and control groups) of at least 2% were tested for overrepresentation in 2 CpG sets of interest:

1. A previously published list of putative metastable epialleles (MEs) identified using the Illumina 450k array (39). There is evidence that DNAm at MEs is established in the early embryo around the time of conception, and these loci display high levels of interindividual variability that may be sensitive to the early nutritional environment (18, 40, 41).
2. A set of CpGs in DMRs in known imprinting control regions (42). Prior evidence exists linking maternal nutrition to methylation status at a number of imprinted loci (9).

Enrichment results were compared against a set of variance-matched control CpGs to assess whether enrichment could be the result of the inherent variability of ME CpGs unrelated to the intervention. Sample permutation was used to derive an estimate for the significance of the enrichment (Supplemental Methods SM4.4).

#### Phenome scan

To gain potential insights into the health relevance of the identified intervention-associated DNAm signals, a phenome scan was performed using the PhenoScanner version 2 database of genome-wide association study (GWAS) associations (43), with the *ESM1* mQTL, rs1423249, as input. PhenoScanner thresholds were set as follows: *P* = 1×10^-4^, *r*^2^ = 0.8, proxies = EUR (European), build = 37.

#### Post hoc sensitivity analyses

A number of post hoc analyses were performed to assess the robustness of the findings from the Gambian EWAS, including DMPs at FDR <5% and those in DMRs. Information on additional sensitivity analyses (Gambia: season of conception interaction analysis; India: maternal BMI and compliance interaction analyses) is provided in Supplemental Methods SM4.5, SM4.6, SM4.7, and SM4.8.

##### SNP probes

The presence of a genetic variant at or near the CpG being measured can influence methylation status or interfere with probe hybridization and incorporation of labeled nucleotides, potentially yielding unreliable intensity measurements. We therefore performed a post hoc sensitivity analysis of the identified DMPs and DMRs to identify CpGs with SNPs within the 50-bp probe sequence from a previously published list of SNPs with minor allele frequency >5% in 1000 Genomes African populations (44).

##### mQTL and genotype × intervention interaction analysis

To determine the extent to which variation in methylation at nutrition-associated loci could be driven by genotype, an initial genome-wide screen for mQTL effects at DMPs and CpGs falling within identified DMRs was performed using the Gene Environment and Methylation (GEM) package from R *Bioconductor*. This uses linear models to test CpGs for direct genetic (mQTL) effects and for genotype × environment (G × E) interactions (45). Processed methylation Beta values, filtered GSA genotypes, and the covariate data (including adjustment and intervention status) were used as inputs for the models. The identified mQTLs were then taken forward for further modeling to help dissect the relative contributions of genotype and intervention. The analysis was repeated using higher-resolution imputed genetic data on chromosome 5 (Supplemental Methods SM4.9).

##### Sex imbalance

We conducted a sensitivity analysis to test whether an observed difference in the numbers of male and female children in each sample group in the Gambian trial could be confounding the reported associations. This was performed for DMPs (FDR <5%). A resampling-based approach was used to simulate 10,000 balanced studies from the observed data and construct 95% CIs for the effect of intervention (Supplemental Methods SM4.10).

## Results

### Epigenome-wide association study

Epigenome-wide screens were performed using the EPIC array to identify DNAm differences in children associated with maternal micronutrient intervention in the 2 cohorts. Details on the cohorts are given in Table 1.

**FIGURE 1:**
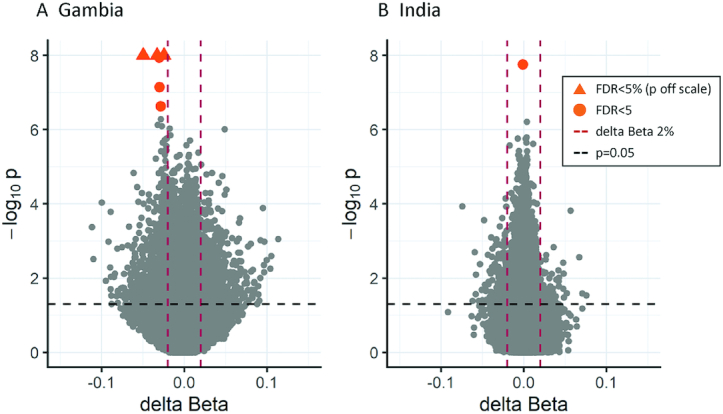
Volcano plots for each cohort of statistical significance against difference in methylation Beta values between intervention and control groups for all CpGs tested. Multiple linear regression models were used for an epigenome-wide association study in both Gambian (A: *n* = 289) and Indian (B: *n* = 686) cohorts. The dashed vertical and horizontal lines indicate thresholds for delta Beta 2% and nominal *P* = 0.05, respectively. Orange circles show differentially methylated positions—significant CpGs with false discovery rate (FDR) <5%; an orange triangle indicates a point falling outside the plot area.

**FIGURE 2:**
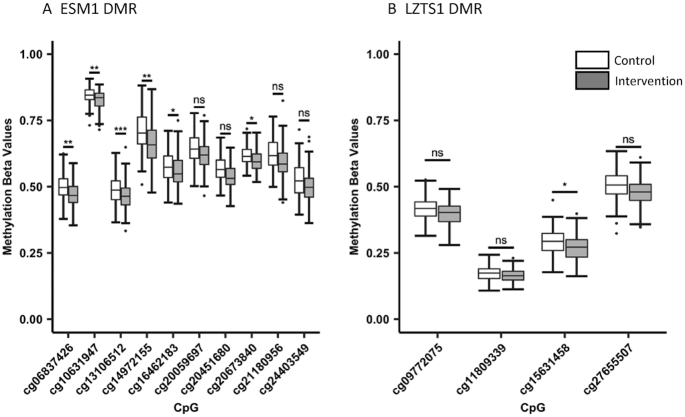
Boxplots showing the distributions of methylation values for the CpGs within the (A) *ESM1* and (B) *LZTS1* DMRs identified in the Gambian epigenome-wide association study regional analysis (*n* = 289). DMRs were identified using *DMRcate* and *comb-p* methods. In *ESM1*, 4 CpGs are also significant differentially methylated positions (DMPs) [false discovery rate (FDR) < 5%]. *LZTS1* contains no significant DMPs. In both regions, the intervention is consistently associated with reduced methylation concentrations. Blank and filled boxes represent control and intervention arms, respectively. CpG site-wise false discovery rates: *FDR < 0.1; **FDR < 1×10^-2^; ***FDR < 1×10^-3^. DMR, differentially methylated regions; ns, not significant.

**FIGURE 3:**
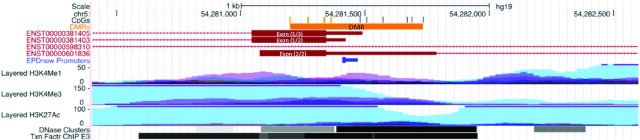
The *ESM1* differentially methylated region (DMR) shown within its genomic context and annotated with regulatory features (data tracks from the University of California, Santa Cruz genome browser). The identified DMR overlaps the transcription start site (TSS), 5′ untranslated region, and first exon of the gene and is in an area of active transcription. The tracks show (in order): the chromosomal location; the CpGs making up the region with differentially methylated positions (false discovery rate <5%) in yellow; the *ESM1* DMR; Ensembl gene predictions (red); predicted promoter location (blue—Eukaryotic Promoter Database v006); histone tail marks H3K4Me1, H3K4Me3, and H3K27Ac indicating active regions of transcription (on 7 cell lines from ENCODE); DNAse hypersensitivity clusters showing open chromatin (125 cell types from ENCODE version 3); and transcription factor ChIP-seq clusters (ENCODE v3).

**FIGURE 4:**
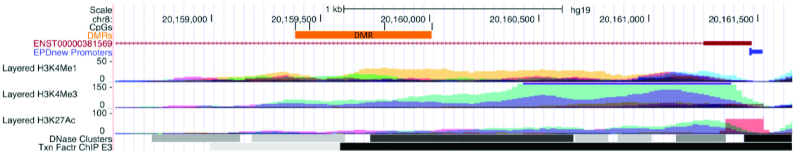
The *LZTS1* differentially methylated region (DMR) within its genomic context and annotated with regulatory features (data tracks from the University of California, Santa Cruz genome browser). The identified DMR is intronic and downstream of a CpG island (green). The tracks show (in order): the chromosomal location; the CpGs making up the region; the *LZTS1* DMR; Ensembl gene prediction (red); predicted promoter location (blue—Eukaryotic Promoter Database v006); histone tail marks H3K4Me1, H3K4Me3, and H3K27Ac indicating active regions of transcription (on 7 cell lines from ENCODE); DNAse hypersensitivity clusters showing open chromatin (125 cell types from ENCODE version 3); and transcription factor ChIP-seq clusters (ENCODE v3).

In the Gambian EWAS, 6 CpGs showed evidence for differential methylation (FDR <5%): cg20451680, cg14972155, cg20673840, cg06837426, cg09612591, and cg05676441 (**Table 2**, **Figure 1**A). All 6 significant DMPs showed decreased methylation associated with the intervention, with effect sizes of −2.5% to −5.0%. The top 4 DMPs are annotated to the endothelial cell-specific molecule 1 (*ESM1*) gene, with all 4 also passing a previously estimated threshold for genome-wide significance (46). The remaining 2 DMPs map to the genes catenin alpha 2 (*CTNNA2*) and cadherin 18 (*CDH18*). A region-level analysis identified a 536-bp long DMR comprising 10 CpGs spanning chromosome 5: 54,281,198 to 54,281,733 mapping to the *ESM1* gene (**Table 3**, **Figure 2**A). This region passed a multiple testing adjusted regional significance threshold (*P* < 0.05) for 2 different DMR methods [*DMRcate* (36) and *comb-p* (37); adjusted *P* = 3.63 × 10^−8^ and 6.06 × 10^−37^ respectively], was the top-ranked region in both, and included all 4 *ESM1* DMPs. The *ESM1* DMR extends over the transcription start site (TSS), 5′ untranslated region (UTR), and first exon of the most highly expressed transcript of the gene and is situated at the junction between alternate transcripts (**Figure 3**). This region is enriched for H3K4Me1, H3K4Me3, and H3K27Ac histone tail marks and increased DNase hypersensitivity in ENCODE cell lines, as well as transcription factor binding (ENCODE ChIP-seq), suggesting it is a regulatory region associated with active transcription. A second DMR, *LZTS1*, consisting of 4 CpGs on chromosome 8 (20,159,382–20,160,009) passed the same FDR threshold with *comb-p* only (adjusted *P* = 3.16 × 10^−11^). This was the second-ranked region in *DMRcate* and also contained a borderline significant DMP (cg27655507; Table 3, Figure 2B, and **Figure 4**). The *LZTS1* DMR maps to an intronic region between exons 1 and 2 of *LZTS1* ∼2 Kb from the TSS. This region shows reduced chromatin accessibility, as evidenced by the absence of DNase hypersensitive sites and the absence of transcriptional factor binding (Figure 4). For both regions, the effect of the intervention consistently decreased methylation across the DMR (Figure 2).

**TABLE 2:** Differentially methylated positions identified in the Gambian intervention epigenome-wide association study^1^

							Beta variability, median (IQR)		
	Probe	Location	Gene	*P* value	FDR	Delta β	Intervention	Controls	Rank
	cg20451680	chr5: 54,281,336	*ESM1*	6.36×10^-10^	5.10×10^-04^	−0.0331	0.53 (0.51, 0.57)	0.56 (0.54, 0.6)	1
	cg14972155	chr5: 54,281,198	*ESM1*	5.10×10^-09^	1.44×10^-03^	−0.0498	0.66 (0.61, 0.71)	0.7 (0.66, 0.76)	2
DMPs	cg20673840	chr5: 54,281,362	*ESM1*	5.39×10^-09^	1.44×10^-03^	−0.0246	0.59 (0.57, 0.62)	0.61 (0.59, 0.64)	3
FDR <5%	cg06837426	chr5: 54,281,271	*ESM1*	1.15×10^-08^	2.30×10^-03^	−0.0303	0.47 (0.44, 0.5)	0.5 (0.47, 0.53)	4
	cg09612591	chr2: 79,519,823	*CTNNA2*	7.13×10^-08^	1.14×10^-02^	−0.0299	0.63 (0.6, 0.66)	0.66 (0.63, 0.7)	5
	cg05676441	chr5: 19,988,800	*CDH18*	2.34×10^-07^	3.13×10^-02^	−0.0286	0.46 (0.43, 0.49)	0.49 (0.46, 0.52)	6
	cg27655507	chr8: 20,159,789	*LZTS1*	5.28×10^-07^	6.05×10^-02^	−0.0283	0.48 (0.45, 0.51)	0.51 (0.47, 0.54)	7
	cg21180956	chr5: 54,281,478	*ESM1*	7.47×10^-07^	7.17×10^-02^	−0.032	0.59 (0.56, 0.63)	0.62 (0.58, 0.67)	8
	cg10631947	chr5: 54,281,668	*ESM1*	9.31×10^-07^	7.17×10^-02^	−0.0176	0.84 (0.8, 0.85)	0.85 (0.83, 0.87)	10
	cg24403549	chr5: 54,281,572	*ESM1*	3.85×10^-06^	1.46×10^-01^	−0.0281	0.5 (0.46, 0.53)	0.52 (0.48, 0.57)	21
CpGs	cg15631458	chr8: 20,160,009	*LZTS1*	4.19×10^-06^	1.46×10^-01^	−0.0242	0.27 (0.23, 0.3)	0.29 (0.26, 0.32)	22
in DMRs	cg13106512	chr5: 54,281,507	*ESM1*	4.80×10^-06^	1.50×10^-01^	−0.0234	0.46 (0.43, 0.49)	0.49 (0.45, 0.52)	24
	cg20059697	chr5: 54,281,687	*ESM1*	2.10×10^-05^	3.01×10^-01^	−0.0236	0.62 (0.58, 0.65)	0.64 (0.61, 0.68)	56
	cg09772075	chr8: 20,159,446	*LZTS1*	6.95×10^-05^	4.16×10^-01^	−0.0192	0.4 (0.37, 0.43)	0.42 (0.39, 0.44)	134
	cg16462183	chr5: 54,281,733	*ESM1*	1.25×10^-03^	7.22×10^-01^	−0.0191	0.55 (0.52, 0.6)	0.57 (0.53, 0.62)	1375
	cg11809339	chr8: 20,159,382	*LZTS1*	6.02×10^-03^	8.40×10^-01^	−0.0074	0.16 (0.15, 0.18)	0.17 (0.15, 0.19)	5730

^1^DMPs passing FDR 5% (above dotted line) and CpGs in DMRs (regional FDR <5%) that did not pass FDR threshold as single probes. Given for each probe are the Illumina probe ID, genomic position (hg19), annotated gene (EPIC manifest), regression *P* value for the effect of intervention group, the FDR-adjusted *P* value, change in mean Beta between intervention and control groups, the Beta value variability [median (IQR)], and finally the rank at which the CpG appears in the list of all tested probes (sorted by *P* value). DMP, differentially methylated position; DMR, differentially methylated region; FDR, false discovery rate.

**TABLE 3:** Differentially methylated regions identified in the Gambian intervention epigenome-wide association study^1^

DMR	Method	Rank	Genomic location	CpGs	Minimum *P*	Region *P* (adjusted)
*ESM1*	*DMRcate*	1	chr5: 54,281,198–54,281,733	10	7.95×10^-47^	3.63×10^-08^
	*comb-p*	1	chr5: 54,281,198–54,281,733	10	1.80×10^-35^	6.06×10^-37^
*LZTS1*	*DMRcate*	2	chr8: 20,159,382–20,160,009	4	2.15×10^-10^	1.81×10^-01^
	*comb-p*	4	chr8: 20,159,382–20,160,009	4	7.35×10^-07^	3.16×10^-11^

^1^Differentially methylated regionswere identified using *DMRcate* and/or *comb-p* methods. Table gives rank, start and end genomic coordinates (hg19), and number of CpGs making up the region. For the *DMRcate* results, minimum *P* is the minimum *P* value for a single site in the region, and region *P* is the Stouffer combined *P* value for the CpGs making up the region. For *comb-p*, minimum *P* is the minimum *P* value for a single site in the region, and region *P* is the multiple testing corrected, regionally adjusted *P* value. DMR, differentially methylated region.

In the Indian EWAS, a single CpG, cg24940138, mapping to the transmembrane protein 106A (*TMEM106A*) gene passed our prespecified 5% FDR threshold for identifying nutrition-associated DMPs. The magnitude of the effect, however, was very small, showing a 0.1% decrease in methylation associated with the intervention (**Table 4**). Sub-significance threshold methylation differences were generally very small, in contrast to the larger observed differences in the Gambian results (Figure 1; Tables 2, 4). Region-level analysis in the Indian EWAS did not reveal any intervention-associated DMRs passing FDR <5%. Overall, based on the findings from CpG-level and regional analyses, the maternal intervention in the Indian trial did not appear to influence DNAm in the children at the sites assessed on the EPIC array.

**TABLE 4:** Top-ranked differentially methylated positions in Indian intervention epigenome-wide association study (by *P* value)^1^

						Beta variability, median (IQR)		
CpG	Location	Gene	*P* value	FDR	delta Beta	Intervention	Controls	Rank
cg24940138	chr17: 41,363,741	*TMEM106A*	1.64×10^-08^	1.32×10^-02^	–0.0012	0.028 (0.026, 0.030)	0.029 (0.027, 0.031)	1
cg19653117	chr2: 32,502,924	*YIPF4*	5.64×10^-07^	2.26×10^-01^	0.0038	0.056 (0.047, 0.070)	0.054 (0.046, 0.066)	2
cg03678138	chr15: 43,029,643	*CDAN1*	1.48×10^-06^	2.56×10^-01^	0.0035	0.077 (0.068, 0.086)	0.074 (0.065, 0.083)	3
cg16663155	chr11: 61,911,154		1.64×10^-06^	2.56×10^-01^	0.0022	0.936 (0.927, 0.943)	0.934 (0.925, 0.940)	4
cg02954365	chr15: 78,585,011	*WDR61;RP11–762H8.1*	1.68×10^-06^	2.56×10^-01^	–0.0073	0.895 (0.880, 0.908)	0.899 (0.887, 0.910)	5
cg12501923	chr17: 73,937,416	*FBF1*	2.06×10^-06^	2.56×10^-01^	0.003	0.048 (0.041, 0.060)	0.046 (0.038, 0.059)	6
cg16962463	chr12: 89,968,675	*RP11–981P6.1*	2.23×10^-06^	2.56×10^-01^	0.0113	0.203 (0.168, 0.247)	0.192 (0.152, 0.236)	7
cg24848351	chr20: 2,622,020	*TMC2*	2.58×10^-06^	2.59×10^-01^	–0.017	0.708 (0.671, 0.755)	0.735 (0.688, 0.770)	8
cg18759102	chr1: 3,100,343		3.56×10^-06^	2.92×10^-01^	0.004	0.058 (0.045, 0.071)	0.053 (0.042, 0.068)	9
cg07438401	chr3: 67,698,057	*SUCLG2*	3.64×10^-06^	2.92×10^-01^	–0.0057	0.891 (0.873, 0.908)	0.898 (0.882, 0.911)	10

^1^Differentially methylated positions passing FDR 5% are indicated above the dashed line. Given for each probe are the Illumina probe ID, genomic position (hg19), annotated gene (EPIC manifest), regression *P* value for the effect of intervention group, the FDR-adjusted *P* value, change in mean Beta between intervention and control groups, the Beta value variability [median (IQR)], and finally the rank at which the CpG appears in the list of all tested probes (sorted by *P* value). FDR, false discovery rate.

We next investigated whether the top hits from the Gambian EWAS, including DMPs at FDR <5% and those in DMRs passing the regional 5% FDR threshold (as listed in Table 2), showed evidence for replication in the Indian EWAS results. None of the Gambian intervention-associated CpGs appeared as top-ranking hits in the Indian EWAS (**Supplemental Table 4**). Two CpGs mapping to *LZTS1*, cg27655507 and cg15631458, were highly ranked (>98th rank percentile), with 3 of 4 CpGs making up the identified DMR showing some consistency of effect direction between the cohorts. However, the methylation differences were small (<1%), and these were not significant at FDR 5%, in keeping with the overall patterns observed in the Indian cohort. Extended DMP and DMR results are available in the **Supplemental Extended Results**.

### DNA methylation at candidate loci

DNAm differences were assessed at 10 candidate loci mapping to 8 genes with prior evidence for sensitivity to maternal nutrition during periconception or in gestation and/or association with relevant health outcomes (Supplemental Table 1) by performing pyrosequencing on samples from both cohorts. No significant intervention-associated regional methylation differences were identified passing FDR <5% in the Gambian or Indian candidates analysis (**Supplemental Table 5, Supplemental Table 6**). However, 2 candidates, *H19* and *PAX8*, did show regional-level and multiple site-level associations at nominal *P* < 0.05 with a consistent effect direction in the Gambian cohort (**Supplemental Table 7**).

### Influence of genetic variation and gene-environment interactions

To investigate the potential influence of genetic variation on nutrition-associated DNAm, we carried out a genome-wide mQTL screen using the GEM package (45), testing all Gambian CpGs within the identified intervention-associated DMRs at regional FDR <5%. Following quality control, 286,552 SNPs with a minor allele frequency >0.05 were obtained for all Gambian samples using the Illumina GSA genotyping array (see Methods). Genotype (G) models identified 7 *ESM1* CpGs showing evidence of mQTL effects at FDR <5%. Methylation status at all 7 CpGs was associated with a single SNP, rs1423249, in the 3′ UTR of the *ESM1* gene, with each variant allele associated with a 0.02–0.04 increase in mean methylation Beta values (**Supplemental Table 8, Supplemental Figure 2**). No other significant mQTLs were identified. G × E (nutritional intervention) interaction models did not identify any interactions passing FDR <5%. A high-resolution mQTL analysis using 405,939 SNPs from imputed genotype data for chromosome 5 did not reveal any additional SNPs driving methylation at *ESM1* beyond the association with rs1423249 already identified.

Additional linear modeling showed that for all 7 *ESM1* CpGs with the *cis* mQTL rs1423249, a G + E model including both genotype (mQTL) and intervention as main effects provided the best fit (higher adjusted *R*^2^ and smaller Akaike information criterion) compared to G, E, or G × E models (**Supplemental Table 9** and Supplemental Methods). For all 7 *ESM1* CpGs, the combined G + E models suggest that intervention and mQTL (allelic) main effects are highly significant and similar in magnitude (**Supplemental Table 10**).

mQTLs may suggest regulatory pathways underlying reported GWAS signals for common diseases (47). We therefore used the single genome-wide significant *ESM1 cis* mQTL to search a database of previously published GWAS associations (43). We found suggestive evidence that rs1423249 and/or associated SNPs in strong linkage disequilibrium are associated with pericarditis and kidney function (**Supplemental Table 11**).

### Enrichment of metastable epialleles and imprinted genes

To assess the presence of subthreshold signals sensitive to periconceptional nutrition (at nominal *P* < 0.05 and absolute methylation Beta difference ≥2%), we tested for enrichment of putative MEs and CpGs associated with genomic imprinting—loci with prior evidence for sensitivity to periconceptional nutrition (9, 18, 39). The results are shown in **Table 5**. In the Gambian EWAS results, there was strong evidence for enrichment of ME CpGs (*P* < 0.0001) and some evidence for enrichment of CpGs associated with imprinted regions (*P* = 0.013), while enrichment was not observed for the variance-matched control set. There was no evidence for enrichment of any of the tested sets in the Indian cohort.

**TABLE 5:** Enrichment analysis for metastable epialleles and loci mapping to imprinted genes^1^

		Data set			
		Gambian		India	
CpG set	*n* in CpG set	*n* subthreshold CpGs in CpG set	Enrichment *P* value	*n* subthreshold CpGs in CpG set	Enrichment *P* value
MEs	1355	80	<1×10^-04^	11	0.97
ICRs	2168	13	1.29×10^-02^	0	1.0
Control: variable CpGs	1355	33	0.15	9	0.68

^1^Both metastable epialleles (MEs) and imprinting-associated CpG sets show evidence for significant enrichment in Gambian epigenome-wide association study CpGs at *P* < 0.05 and delta Beta ≥2%. ICRs, imprinting control regions—CpGs associated with genomic imprinting (see Methods).

### Sensitivity analyses

The 6 DMPs identified in the Gambian EWAS were robust to the batch/technical variable modeling strategy, with CpGs mapping to *ESM1* consistently in the top-ranked CpGs (**Supplemental Table 12**). No DMPs contained common African SNPs either at the CpG or within 50 bp that might interfere with probe hybridization (see Methods). Further, methylation differences at these DMPs were not driven by sex imbalance between intervention and control groups (**Supplemental Table 13**). Last, there was no evidence for an interaction between the intervention and Gambian season of conception (SoC) at the 6 DMPs tested (**Supplemental Table 14**), a nutrition-related exposure previously shown to predict DNAm in Gambians (17, 18). A similar analysis for candidate loci also found no evidence for a SoC interaction effect (**Supplemental Table 15**).

We did not identify an intervention effect in the Indian cohort, as evidenced by the observed small methylation differences, the presence of only a single DMP passing FDR <5% and absence of any DMRs, and the observation that the top-ranking CpGs were not replicated using different modeling strategies (data not shown). A number of sensitivity analyses were performed to test other possible explanations for this result (Supplemental Methods). First, a positive control analysis with maternal BMI, an exposure with prior evidence of an effect on offspring methylation, showed an effect size distribution indicative of a nonnull effect following the same analysis pipeline (**Supplemental Figure 3**, Figure 1B). A previous study in the same Indian cohort revealed an interaction between intervention and maternal BMI on birth weight of children (25). We therefore tested for interaction between intervention and maternal BMI, as well as for differences in intervention effects on DNAm between maternal BMI strata. We did not find any evidence for either a significant interaction between maternal BMI and intervention or differences in size of intervention effect between maternal BMI subgroups (**Supplemental Figure 4**). We also found no evidence of an interaction between intervention and compliance (**Supplemental Figure 5**; Supplemental Results) or of any significant effect of the intervention in an analysis restricted to fully compliant women (**Supplementary Table 16**; Supplemental Results).

### Technical validation and cross-tissue replication

Technical validation of *ESM1* and *LZTS1* methylation was performed in 92 samples from both cohorts by pyrosequencing all 4 significant DMPs located in the *ESM1* DMR (cg14972155, cg06837426, cg20451680, cg20673840; all FDR <5%) and the most significant DMP in the *LZTS1* DMR (cg27655507; FDR ∼5%) (further details in Supplemental Methods, pyrosequencing primers in **Supplemental Table 17**). We found very good agreement between the methylation values obtained on the EPIC array and pyrosequencing platforms, with Spearman's rank correlation coefficient in the range of 0.8 to 0.9, all *P* < 2.2 × 10^−16^ (**Supplemental Figures 6–7**).

To assess tissue specificity of DNAm and to detect patterns of systemic interindividual variation in DNAm suggestive of putative metastability, we performed bisulfite pyrosequencing for DNA derived from buccal and blood cells in 92 samples from the Gambian cohort at the *ESM1* and *LZTS1* loci listed above. We found blood and buccal DNAm was correlated across all tested loci (Spearman's ρ = 0.36–0.50; *N* = 75–89, all *P* < 0.001) (**Supplemental Table 18, Supplemental Figures 8–9**).

## Discussion

We report the first findings from the EMPHASIS study (24) in which we investigated the impact of maternal nutrition on DNA methylation patterns in children whose mothers participated in 2 independent micronutrient supplementation RCTs in India and The Gambia. We identified a number of loci showing altered DNAm in children aged 7–9 y in response to maternal micronutrient supplementation in the Gambian cohort. In contrast, no robust DNAm differences were identified in children aged 5–7 y in the Indian cohort.

The Gambian EWAS using the Illumina EPIC array identified 6 DMPs passing a previously specified FDR threshold of 5%, all of which showed decreased methylation in the intervention group compared with controls. Regional (DMR) analysis confirmed a robust association at a region containing 10 CpGs mapping to the *ESM1* gene, overlapping with the TSS, 5′ UTR, and first exon of the most highly expressed transcript. A second region made up of 4 CpGs mapping to an intronic region of *LZTS1* ∼2 Kb from the TSS was the top-ranked significant DMR with *comb-p* and the second-ranked (nonsignificant) DMR using *DMRcate*. Increased DNAm in promoter regions is typically associated with gene silencing, whereas in the gene body, the relation can vary depending on context (48). Further work is required to determine the relation between the identified intervention-associated DMPs/DMRs and gene expression.

The endothelial cell-specific molecule 1 (*ESM1* or endocan) gene encodes a proteoglycan secreted by endothelial cells and preferentially expressed in lung and kidney. Molecular functions of the protein include hepatocyte growth receptor binding and insulin-like growth factor binding, and associated biological functions include angiogenesis and regulation of cell growth (49). Several studies have demonstrated a link between serum concentrations of *ESM1* and hypertension and cardiovascular disease (50), as well as renal damage (51), suggesting it may have clinical utility as an inflammatory biomarker (50–52). This gene has also been linked to obesity (53) and diabetes through diabetes-mediated endothelial dysfunction (54) and subclinical atherosclerosis in patients with type 2 diabetes (55). Perhaps most pertinent to the present study, *ESM1* has been associated with both maternal and neonatal factors, including gestational diabetes mellitus (GDM) (56), preeclampsia (57, 58), and birth weight (57). Hjort et al. (56) identified a number of CpGs mapping to *ESM1* with evidence of differential methylation in children associated with maternal GDM status, with some also independently associated with maternal prepregnancy BMI. This lends support to our finding that *ESM1* methylation may be sensitive to early maternal factors related to nutrition. Taken together, the above findings point to a possible relation between maternal nutritional exposures, regulation of *ESM1*, and endothelial dysfunction, which could be relevant to later health and disease risk. We have the potential to explore some of these putative links in the next stage of the EMPHASIS study, in which we will examine associations between DNAm and phenotypic outcomes in the children.

Leucine zipper tumor suppressor 1 (*LZTS1*) is a ubiquitously expressed tumor suppressor involved in the regulation of cell growth. It has previously been associated with esophageal and other cancers in humans (59), whereas an expression study in mouse and chick embryos has suggested it may have a role in neuronal development (60). We believe this is the first putative link between *LZTS1* and a nutritional exposure in humans, although further studies are required to determine relevance if any to development and childhood phenotypes. A further 2 FDR significant DMPs were identified that mapped to cadherin 18 (*CDH18*) and catenin alpha 2 (*CTNNA2*). These genes code for cell adhesion proteins involved in synaptic adhesion and axon growth and guidance during nervous system development (61). *CTNNA2* has previously been associated with a number of phenotypes, including Alzheimer disease (62) and blood pressure (63), whereas *CDH18* has similarly been associated with blood pressure traits, including hypertension (64).

In the Indian EWAS, 1 DMP mapping to *TMEM106A* passed FDR <5%. However, observed Beta differences at this DMP and at other subthreshold loci were very small (most <1%), and this, together with the nonreproducibility of the results using different modeling strategies, suggested no evidence for an effect of intervention on methylation in this cohort. This conclusion was supported by sensitivity analyses and by the findings from the candidate locus analyses. Consistent with this, significant DMPs from the Gambian analysis did not replicate in the Indian EWAS results.

A parallel analysis investigated associations with the nutritional intervention at loci not on the EPIC array but with prior evidence of links to maternal nutrition and/or health outcomes collected as part of the EMPHASIS study (24). None of the candidates in either cohort showed significant regional DNAm differences associated with the intervention at FDR <5%. However, in the Gambian candidates, we found consistent associations (nominal regional *P* < 0.05) across all measured CpGs in 2 regions: 1) a region proximal to the imprinting control region of *H19*, a gene involved in fetal growth and development, and 2) *PAX8*—a previously identified human metastable epiallele. Decreased methylation at *H19* has been associated with folic acid supplementation taken before and during pregnancy (65). Previous studies in The Gambia have found increased methylation of *PAX8* associated with rainy season conceptions (66), an exposure associated with an increase in certain maternal 1-C micronutrient concentrations. For both regions, the direction of the effects across the regions were consistent with these previous findings.

Many possible reasons could explain why DNAm differences associated with the intervention were not identified in the Indian cohort. This could reflect the much lower micronutrient concentrations in the food-based supplement used in the Mumbai trial compared with the multiple micronutrient tablet used in the Gambian trial, the different combinations of nutrients, and/or their different bioavailability. The 2 populations also have contrasting nutritional and genetic backgrounds, and there is the potential influence of different postnatal environments. Intercohort differences in child age and in other maternal factors, such as lower maternal height and age in the Indian participants, might also play a role (24). The timing of the intervention differed between the trials: stopping in early pregnancy in the Gambian trial while continuing until delivery in the Indian trial. In the Gambian tablet-based trial, compliance was monitored using tablet counts and was high (88% of all tablets were eaten in the intervention group and 86% in controls). In the Indian trial, women were offered 1 food-based supplement/snack per day 6 d per week, and full compliance was defined as 3 or more per week; only 43% in the intervention group and 58% in the control group achieved this. Compliance in the Mumbai trial was therefore low by the usual standards of an RCT. The supplement was started preconceptionally to cover the earliest days and weeks of pregnancy, but this meant that women often took the supplement for many months before conceiving. Food-based interventions are particularly challenging in this respect because food is a personal choice, whereas people are used to taking tablets without expecting any aesthetic qualities from them.

We have previously reported that despite low compliance, the Indian intervention significantly reduced gestational diabetes (28) and increased birth weight (25). The Gambian intervention had no effect on birth weight but improved 1 measure of placental function (uterine-artery resistance index) (26). We did not investigate links between intervention-associated CpGs (with DNAm measured in mid-childhood) and these previously published intervention-associated maternal and neonatal birth outcomes because it would be difficult to discount reverse causation effects. It is possible (in both cohorts) that some DNAm differences between intervention and control groups were present earlier and had decreased or disappeared by mid-childhood (67). We were not able to investigate such longitudinal changes in DNAm as neonatal samples were not collected in the original studies.

DNAm can be influenced by genotype, and genetic variation may therefore be an important confounder in observed associations (68), although this is unlikely given the randomized design of this study. A genome-wide mQTL analysis of Gambian intervention-associated CpGs provided evidence that methylation status of *ESM1* is independently influenced by the intervention and by genotype in *cis*. There is existing evidence that the identified *cis-*mQTL, rs1423249, is also a *cis*–expression quantitative trait locus for *ESM1* in human lung tissue (69), suggesting this variant might influence *ESM1* expression through its effect on methylation at the *ESM1* DMR. Identification of mQTLs can also provide insights into regulatory pathways underlying GWAS signals for common diseases (47). We therefore explored phenotypic associations at the identified *cis*-mQTL influencing multiple CpGs at the *ESM1* DMR and found suggestive associations with pericarditis and kidney function. Further support for these links will require functional work to assess the relation between *cis-*genotype, methylation at the *ESM1* DMR, gene expression, and relevant biological pathways.

Findings from human and animal studies suggest that MEs and CpGs mapping to imprinted genes may be particularly sensitive to maternal periconceptional nutrition (9, 41). We looked for evidence of enrichment of loci mapping to MEs and imprinted regions using a less conservative significance threshold to capture potential subthreshold signals not detected in DMP and DMR analyses. We found strong evidence for enrichment of MEs and weaker evidence for enrichment of CpGs mapping to imprinting control regions in the Gambian cohort. Strong enrichment for putative MEs provides further evidence that these loci, identified in a previous screen in independent samples, may be sensitive to periconceptional maternal nutrition and thus bear the hallmarks of metastability. Observed correlations of methylation between blood and buccal cell methylation at *ESM1* and *LZTS1*—cells derived from different germ layers—combined with increased interindividual variation at these loci are again a hallmark of MEs. This further indicates that the supplement may be exerting its influence in the first days following conception, when the developing embryo's epigenome undergoes extensive remodeling (40).

Although we might expect the provision of micronutrients relevant to 1-C metabolism to increase availability of methyl donors and essential cofactors, thereby increasing DNAm, we found the reverse. Evidence on relations between nutritional exposures and DNAm patterns is inconsistent, varying by supplemental form, timing of exposure, genetic and nutritional background, and the genetic loci investigated (9). Although it is not possible to fully disentangle the effect of any single micronutrient from the multiple micronutrient interventions studied here, it is notable that 2 recent RCT-based studies found an inverse relation between maternal folic acid supplementation during pregnancy and offspring DNAm (21, 22). Given that the daily UNIMMAP supplement used in the Gambian intervention contains 400 µg folic acid, alongside 14 other micronutrients, it is possible this component may be driving the inverse relation observed.

The EMPHASIS study targets Indian and Gambian populations with poor nutrition and increased rates of morbidity compared with high-income countries. The elucidation of epigenetic mechanisms linking maternal nutritional exposures to poor health outcomes therefore has the potential to make an impact in areas with a high burden of disease. Importantly, both cohorts follow up on randomized controlled intervention trials, thereby strengthening causal inference by minimizing the potential for confounding and bias evident in observational studies. Alternative causal pathways are possible, however, notably reverse causation, whereby the nutrient intervention influences a postnatal phenotypic trait, which in turn affects DNAm at associated loci. These issues are particularly challenging in the context of epigenetic studies as DNAm has been associated with a wide range of exposures and outcomes, including many relating to maternal effects during pregnancy and prepregnancy. In the next stage of the EMPHASIS study, analyses of DNAm-phenotype associations in conjunction with causal analysis techniques such as Mendelian randomization may shed light on this. Were causal links to be established, this would provide opportunities to gain insights into the efficacy of periconceptional nutritional interventions for improving health in the next generation.

Limitations of the study, many of which we discuss in more detail above, include the relatively small samples sizes, particularly in the Gambian cohort, and the lower multiple micronutrient input in the Indian food-based supplement. Also, neonatal DNA samples were not available, so we were unable to explore the stability of DNAm signatures over time or the possible attenuation of intervention-associated DNAm with age. Finally, the EPIC array necessarily offers limited coverage of the human methylome, leaving us unable to detect nutrition-DNAm associations at the majority of genomic methylation sites, including many putative nutrition-sensitive sites identified using whole-genome bisulfite sequencing screens (40).

In conclusion, our evidence suggests that micronutrient supplementation of Gambian mothers in the periconceptional period may alter children's DNAm, measured at 7–9 y. Further studies are required to link the observed methylation changes to differences in gene expression and to assess links to health-relevant outcomes.

## Supplementary Material

nqaa193_Supplemental_File

## Notes

Full list of authors and affiliations for authors belonging to the EMPHASIS Study Group are given at the end of the main text.

AS, SS, and PI, joint first authors.

CHDF, GRC, and MJS, joint last authors.

MMNP supported by the Wellcome Trust, Parthenon Trust, ICICI Bank Ltd., UK Medical Research Council (MRC), and UK Department for International Development (DFID) under the MRC/DFID Concordat. Children's follow-up funded by MRC (MR/M005186/1). PMMST supported by MRC (U1232661351, U105960371, and MC-A760-5QX00) and DFID under the MRC/DFID Concordat, and other members of the Gambian team are supported by MRC grants U105960371, U123261351, and MR/M01424X/1. The EMPHASIS study is jointly funded by MRC, DFID, and the Department of Biotechnology (DBT), Ministry of Science and Technology, India under the Newton Fund initiative (MRC grant MR/N006208/1 and DBT grant BT/IN/DBT-MRC/DFID/24/GRC/2015–16). The funding bodies played no role in the design of the study and collection, analysis, interpretation of data, or writing of the manuscript.

EPIC data have been placed on the server of the CSIR–Centre for Cellular and Molecular Biology in Hyderabad and can be accessed with permission from the authors. Bespoke code written for the analysis is available at https://github.com/asaffa/EMPHASIS.

Supplemental Tables 1–18, Supplemental Figures 1–10, Supplemental Methods, and Supplemental Extended Results are available from the “Supplementary data” link in the online posting of the article and from the same link in the online table of contents at https://academic.oup.com/ajcn/.

Abbreviations used: 1-C, 1 carbon; DMPs, differentially methylated positions; DMRs, differentially methylated regions; DNAm, DNA methylation; EMPHASIS, Epigenetic Mechanisms linking Preconceptional nutrition and Health Assessed in India and Sub-Saharan Africa; EWAS, epigenome-wide association study; FDR, false discovery rate; GDM, gestational diabetes mellitus; G × E, genotype × environment; GEM, gene environment and methylation; GSA, global screening array; GWAS, genome-wide association study; HWE, Hardy-Weinberg equilibrium; MAF, minor allele frequency; ME, metastable epiallele; MMNP, Mumbai Maternal Nutrition Project; MRC, Medical Research Council; mQTL, methylation quantitative trait locus/loci; PMMST, Periconceptional Multiple Micronutrient Supplementation Trial; QC, quality control; RCT, randomized controlled trial; RNI, Reference Nutrient Intake; SNP, single-nucleotide polymorphism; SoC, season of conception; TSS, transcription start site; UNIMMAP, United Nations International Multiple Micronutrient Preparation; UTR, untranslated region.
