# Supplementary material for: Effect of maternal preconceptional and pregnancy micronutrient interventions on children's DNA methylation: Findings from the EMPHASIS study
Source: Am J Clin Nutr. 2020 Sep 5;112(4):1099–113. doi: 10.1093/ajcn/nqaa193 (PMC7528567; doi:10.1093/ajcn/nqaa193)
Supplement: nqaa193_Supplemental_File [file nqaa193_supplemental_file.docx]

**Effect of maternal preconceptional and pregnancy micronutrient interventions on children’s DNA methylation: findings from the EMPHASIS study**

Saffari et al.

**Online Supplemental Material**

Supplemental Methods

**SM1 Samples**

**SM1.1 DNA isolation**

**SM2 Epigenome-wide DNA methylation**

**SM2.1 Infinium MethylationEPIC BeadChip Processing**

**SM2.2 Sample Randomisation**

**SM2.3 Quality Control (QC)**

**SM2.4 Normalization**

**SM2.5 Technical validation by pyrosequencing**

**SM3 Genotyping**

**SM4 Epignome-wide Association Study (EWAS)**

**SM4.1 Site-level differential methylation analysis**

**SM4.2 Control of genomic inflation (Indian cohort only)**

**SM4.3 Region level differential methylation analysis
SM4.4 Enrichment analysis**

**SM4.5 Positive control EWAS (Indian cohort only)**

**SM4.6 Maternal BMI interaction EWAS (Indian cohort only)**

**SM4.7 Compliance (Indian cohort only)**

**SM4.8 Season of conception interaction (Gambian cohort only)**

**SM4.9 mQTL analysis (Gambian cohort only)**

**SM4.10 Sex balance sensitivity (Gambian cohort only)**

**SM5 Candidate genes**

**SM5.1 Site-level differential methylation analysis**

**SM5.2 Region level differential methylation analysis**

**SM5.3 Season of conception interaction (Gambian cohort only)**

Supplemental Results

Supplemental Figures

Supplemental Tables.

Supplemental Methods

**SM1 Samples**

**SM1.1 DNA isolation**

DNA isolation was carried out in batches of 16 samples using QiaAmp Blood Midi kits. STR profiling was performed on one batch of samples, selected at random, to ensure there was no cross contamination between the samples.

**SM2 Epigenome-wide DNA methylation**

**SM2.1 Infinium MethylationEPIC BeadChip Processing**

Samples required a starting genomic DNA concentration of at least 25 ng/uL, which led to the exclusion of 11 Indian and 3 Gambian samples, leaving 698 and 293 samples for methylation profiling in Indian and Gambian cohorts respectively. Next, 500 ng of genomic DNA was modified by treatment with sodium bisulfite using the EZ-96 DNA Methylation-Gold kit protocol (Zymo Research; Irvine, CA) and subsequently transferred to MSA4 plates for whole genome amplification. Samples were distributed on the plates following a balanced, randomized design (see below). Following overnight amplification, the prepared libraries were hybridized on to beadchips. Eight samples were loaded onto each of the Infinium MethylationEPIC BeadChips. A total of 64 samples (8 per chip) were processed at a time, with a total of 17 batches processed in total. After overnight hybridization the bead chips were processed for single base extension, staining and washing following the manufacturer’s instructions. Finally, vacuum dried bead chips were scanned using the Illumina iScan.

**SM2.2 Sample Randomisation**

Samples were arranged on the MSA4 plates in a randomized, balanced design. The Optimal Sample Assignment Tool package from R/Bioconductor was used to randomly distribute samples in groups of biological interest into different batches, to reduce the possibility of confounding between batches and biological variables of interest ^1^. Samples were randomized and balanced with respect to intervention (both cohorts) and season of conception (Gambian cohort only) firstly at the level of plate, and then across plate columns (corresponding to the chips which the samples were subsequently loaded on to).

**SM2.3 Quality Control (QC)**

GenomeStudio was used to perform an initial assessment of the data quality. Metrics corresponding to the different classes of control probes on the array were inspected measuring bisulphite conversion efficiency, probe specificity, hybridization, target removal, staining, base extension, and other aspects of the assay. All metrics were found to be within expected ranges, and indications were that all of the arrays had produced high quality measurements. In addition, to check consistency between the batches, included Illumina control DNA was compared. No significance differences between batches were observed.

Next, the data was imported into *R* and *meffil* ^2^ was used for further QC and preprocessing, following the standard pipeline. Firstly, methylation predicted sex (as determined by the difference between median methylation of X and Y chromosome probes) was used to check sample labels and to detect and remove sex outliers, defined as those > 5 SDs from the mean. This led to the identification and removal of 5 sex mismatches in the Indian dataset, whilst no samples were removed from the Gambian dataset. Next, the median methylated to unmethylated signal from the control probes was used to identify outlier samples and remove any that were > 3 SDs from the fitted regression line. This resulted in the removal of 7 Indian and 4 Gambian samples. Next, probe filtering was performed to remove probes with detection p-value > 0.01 and number of beads < 3 across more than 10% of samples. This lead to the removal of 1494 probes from the Indian and 2635 probes from the Gambian dataset. Finally, additional probe filtering was performed to exclude unreliable probes previously found to be multi-mapping or cross-hybridizing ^3^, and probes mapping to X and Y chromosomes. This led to the removal of 61,523 probes from the Indian and 61,225 probes from the Gambian datasets.

**SM2.4 Normalization**

For normalization of the array data we followed the standard meffil approach, which performs dye-bias and background correction using the normal-exponential out-of-band (*noob*) method ^4^ and an implementation of functional normalization ^5^- a quantile normalization based approach utilizing principal components derived from control probes, to reduce between-array variability.

*Meffil* includes the facility to specify the number of principal components (PCs) to include in functional normalization to minimize unexplained residual variance. Scree plots were produced and inspected to determine the PC at which the proportion of variance explained by subsequent components appeared to reach a limit, which was then used as the parameter for functional normalization. For both datasets, 20 control probe PCs were used.

**SM2.5 Technical validation by pyrosequencing**

Pyrosequencing was used to validate intervention-associated loci at Endothelial cell specific molecule 1 (*ESM1*) and Leucine-zipper-like transcriptional regulator 1 (*LZTS1*) regions using subsamples comprising 92 samples each from the Gambian and Indian cohorts. For the Gambian cohort, samples were selected from those where buccal swabs were also available. For both cohorts samples were selected to ensure coverage over the range of possible methylation values for each site. Assays were designed using the PyroMark Assay Design Software ver. 2.0.1.15 and sequencing performed using the PyroMark Q96 MD pyrosequencer (both Qiagen, Hilden, Germany). Polymerase chain reaction (PCR) conditions were optimized to produce a single, robust amplification product, and pyrosequencing was then performed as per the manufacturer’s instructions. Fully methylated (100%) and non-methylated (0%) standards (Zymo Research, California, USA) and a positive control DNA were used to demonstrate consistent methylation across all batches.

**SM3 Genotyping**

A total of 200 ng of genomic DNA was used and processed as per the manufacturer’s instructions. Arrays were processed 24 samples per chip and eight chips per batch. Global Screening Array (GSA) chips were scanned on an Illumina iScan machine and output files generated in intensity data file (.idat) format. Samples required a call rate > 95% to be taken forward for analysis, which in the Gambian data led to the exclusion of 5 samples and went through preliminary QC. All filtering ,GenTrain Score, Cluster separation and other parameters in the Genome studio genotype module were used as per previous recommendations ^6^. After GenomeStudio QC, genotype data for 290 Gambian samples and 642,824 single nucleotide polymorphisms (SNPs) were taken forward for further analysis.

**SM4 Epignome-wide Association Study (EWAS)**

**SM4.1 Site-level differential methylation analysis**

For single-site level differential methylation analysis, a linear modelling based approach was used. Firstly, to account for batch or technical effects, three different sets of latent variables were generated, using Principal Components Analysis (PCA), Surrogate Variable Analysis (SVA) ^7^ and Independent Surrogate Variable Analysis (ISVA) ^8^, using a subset of the 200,000 most variable CpGs to reduce computation time. The decision on which latent variables to include in the final model was informed by inspecting correlations/associations between constructed variables and known biological and technical variables, and examining scree plots showing methylation variance explained, to ensure adjustment for cell composition and batch/technical effects. These steps were carried out independently for each cohort.

For regression modelling, the *limma* package was used - a linear regression-based method using a moderated version of the t statistic to improve array wide standard error estimation for identifying significant differences between sample groups.

The following five models were tested:

All:

*M ~ slide + array +Bcell + CD4T + CD8T + Eos + Mono + NK + Neu + sex + childs_age + season_of_conception (Gambian only) + intervention*

All w/o BCCs:

*M ~ slide + array + sex + childs_age + season_of_conception (Gambian only) + intervention*

PCs:

*M ~ PCs + childs_age + season_of_conception (Gambian only) + intervention*

SVs:

*M ~ SVs + childs_age + season_of_conception (Gambian only) + intervention*

ISVs:

*M ~ ISVs + plate_well (Indian only) + sex (Gambian only) + childs_age + season_of_conception (Gambian only) + intervention*

Where M is methylation status as M-values (obtained by logit transformation of the Beta values). In the Gambian models season of conception was also included, as this is a known DNAm-associated exposure in this population ^9,10^.

Model stability and assumptions were assessed informally by: a) visual inspection of QQ plots and p distribution histograms; b) lambda value calculation (ratio of the median observed chi square value distribution to the expected) to assess levels of genome-wide inflation in the test statistics; c) assessment of variance inflation factors to identify collinearity in model predictors; and d) assessment of inter-model consistency for top-ranking (by p-value) differentially methylated positions (DMPs). On this basis, the final models selected were the ISVs model for the Indian EWAS (using 13 ISVs), and the PCs model for the Gambian dataset (using 14 PCs). QQ plots for final models are shown in Supplemental Figure 10. Sensitivity of coefficients and p-value rankings to model choice for the identified DMPs is summarised in Supplemental Table 10.

**SM4.2 Control of genomic inflation (Indian cohort only)**

The metrics and plots generated for the primary analysis model using ISVs indicated the presence of genomic inflation (lambda = 1.15). Epigenome-wide studies are often subject to test statistic inflation and bias, potentially leading to an increased false positive rate. To control for inflation we used the *bacon* package in R ^11^ which indicated our data comprising 803,120 test-statistics had an estimated bias of -0.26 and lambda of 1.04.

**SM4.3 Region level differential methylation analysis**

In addition to site-level analysis, region-level analysis was performed to identify differentially methylated regions (DMRs). The rationale for investigating regional methylation signals is as follows: i) spatially proximal CpG sites show correlated methylation status (comethylation) up to around 1-2 Kb ^15^ ii) combining evidence from multiple (potentially comethylated) CpGs may give a more robust signal, iii) groups of proximal and/or correlated CpGs might have functional relevance (e.g. CpG islands). Multiple methods for identifying differentially methylated regions exist, and these typically rest on different assumptions. We used two different methods:

1. *DMRcate* ^16^ from the *Bioconductor* package *missMethyl* ^17^. This uses the moderated t statistics from the site-level *limma* results, applies a Gaussian kernel to smooth these within a specified window, models the smoothed statistics using the method of Satterthwaite, applies Benjamini - Hochberg False Discovery Rate (BH FDR) correction to the values at each location and then agglomerates nearby significant sites into regions. The following default parameters were used: bandwidth/window size: λ = 1000, scaling factor for bandwidth: C = 2. Significant regions were defined as those passing FDR < 5%.
2. *comb-p* (provided as a *Python* command line tool) ^18^. This spatially adjusts p values based on their autocorrelation using the Stouffer-Liptak-Kechris (SLK) method, identifies enriched regions of adjacent small p values, and reports the spatially adjusted p for the region as well as a Sidak multiple testing corrected value. The following parameters were used: --seed 5e-2 --dist=1000.

Results from each method were compared to check for consistency.

**SM4.4 Enrichment analysis**

EWAS results for both cohorts were tested for enrichment of specific CpG sets of interest, setting a cutoff of nominal p < 0.05 and delta beta >= 2% to include top ranked CpGs as well as potential subthreshold signals. Two pre-specified lists of CpGs were tested : i) a previously curated set of metastable epialleles (MEs) on the 450k array ^10^, ii) CpGs in known imprinted DMRs ^20^. Finally, a set of highly variable CpGs was used as a control. To obtain the control set, non-ME CpGs were first placed into bins corresponding to the deciles of the ME variance distribution, CpGs were then randomly selected from each bin to make up a set of CpGs of the same size and approximate variance distribution as MEs.

A self-contained geneset enrichment hypothesis was tested using sample permutation. The following algorithm was used (based on ^21^) : i) shuffle intervention status labels for the subjects 10,000 times, ii) for each permutation, run *limma* linear regressions (with models as previously specified for the main EWAS analysis) for all the CpGs in each of the sets of interest, iii) record the number of differentially methylated CpGs at p < 0.05; absolute beta difference >= 2%), iv) calculate permutation p value as the proportion of simulations where the number of DMPs in the set was equal to or greater than the experimentally observed value.

**SM4.5 Positive control EWAS (Indian cohort only)**

To increase confidence in the null finding for the Indian intervention EWAS, we performed a positive control analysis using maternal body mass index (BMI) as an exposure, since there is some prior evidence that this influences child methylation ^12^. The expectation was that by using the same data and analysis strategy but with a different exposure (maternal BMI), we should observe an effect size distribution different to that observed in Figure 1b. A similar model was used to the main intervention EWAS, with surrogate variables (ISVs) used to adjust for cell composition and batch/technical effects, but with maternal BMI as the variable of interest:

*M ~ ISVs + sex + maternal_BMI*

**SM4.6 Maternal BMI interaction EWAS (Indian cohort only)**

The original Mumbai trial demonstrated an effect of the nutritional intervention on birthweight that was moderated by maternal BMI ^13^. We therefore considered the possibility of maternal BMI confounding or mediating/moderating the relationship between the intervention and the child’s DNA methylation. We examined whether there was a significant interaction between maternal BMI and intervention group:

*M ~ ISVs + plate_well + childs_age + maternal_BMI + intervention + maternal_BMI: intervention*

Secondly, median maternal BMI (19.5) was calculated and child methylation data was segregated based on maternal BMI less than 19.5 and greater than 19.5, and differential methylation analysis performed for both subgroups using the model:

*M ~ ISVs + plate_well _ childs_age + subset_maternal BMI + intervention*

**SM4.7 Compliance (Indian cohort only)**

Compliance is an important consideration in intervention studies. In the Indian trial, only around 50% of mothers overall complied fully with the intervention (took 3 or more supplements per week during the period 3 months prior to and 2 weeks after conception). We therefore examined (1) whether there was evidence for an interaction between maternal compliance and intervention group on the child’s DNA methylation and (2) secondly in a stratified analysis (children of compliant mothers and children of non-compliant mothers) whether there was any evidence that the effect of the intervention on DNAm differed between compliant and non-compliant groups.. The following model was used to test for interaction:

*M ~ ISVs + covariates + intervention + compliance status + intervention:compliance status*

Stratified analysis was performed separating children whose mothers were compliant and those who were not compliant and using the same model for each subgroup:

*M ~ ISVs + plate_well + childs_age + intervention*

**SM4.8 Season of conception interaction (Gambian cohort only)**

In the Gambia, season of conception (SoC) is an exposure that is known to predict methylation status. Since SoC may serve as a proxy for significant differences in methyl donors and other micronutrients ^14^, we investigated potential interaction with SoC by performing a sensitivity analysis for CpGs at FDR < 5%, using the follow regression model:

*M ~ PCs + childs_age + season_of_conception + intervention + season_of_conception : intervention*

**SM4.9 mQTL analysis (Gambian cohort only)**

Gambian EWAS CpGs making up the DMRs were tested for mQTL effects using the Gene Environment and Methylation (*GEM*) package from Bioconductor ^19^ which adapts the computationally efficient *Matrix eQTL* package for testing the interplay between genotype, methylation, and environment. Linear models are used to test the independent effect of genotype on methylation (G model), environment on methylation (E model), and then the interaction between gene and environment on methylation (G x E model) - see ^19^ for further details. The following were used as inputs to *GEM*:

1. EPIC array methylation beta-values for 14 CpGs which included all those within the DMRs at FDR < 5%,
2. cleaned and filtered GSA genotype data for 286,552 SNPs (coded as allelic dosages)
3. phenotype data - with intervention as the “environmental” variable of interest, and the following covariates for adjustment: PCs, childs_age, season_of_conception

The following models were tested by GEM:

G model:

*Beta ~ PCs + childs_age + season_of_conception + genotype*

E model:

*Beta ~ PCs + childs_age + season_of_conception + intervention*

G x E model:

*Beta ~ PCs + childs_age + season_of_conception + genotype + intervention + genotype:intervention*

A single mQTL (FDR < 5%) was identified by the *GEM* G model, and this was taken forward for further statistical modelling to asses the combined effects of genotype and intervention (and their potential interaction) on methylation. The same G, E, and G x E models as before but with the addition of G + E (below) were assessed by comparing their adjusted R^2^ values and AIC (Akaike Information Criterion).

G + E model:

*Beta ~ PCs + childs_age + season_of_conception + genotype + intervention*

**SM4.10 Sex balance sensitivity (Gambian cohort only)**

There were differing numbers of male and female births in each arm of the Peri-conceptional Multiple Micronutrient Supplementation Trial (PMMST) trial, which was further skewed by unequal dropout rates in children enrolling in the EMPHASIS follow-up study. Sex ratios for samples analysed in the EWAS were: 0.86 (M:F) in the intervention group and 1.60 (M:F) in the control group (chi-sq p < 0.02). To test whether skewed sex ratios could be influencing the results, a resampling based approach was used to simulate a balanced study. Briefly, subjects were randomly sampled without replacement 10,000 times by fixing the male to female ratio in both arms at 1:1 giving sample sizes of 232 : 116 males and 116 females (the largest possible sample allowing equally sized groups without replacement). Linear regressions for the CpGs at FDR < 5% were performed for each simulation, and the results used to construct 95% confidence intervals for the estimated coefficients for the effect of intervention on methylation. The observed (empirical) coefficients for the CpGs were then compared to these intervals to determine if the estimated coefficients were influenced by skewed sex ratios.

**SM5 Candidate genes**

**SM5.1 Site-level differential methylation analysis**

Following preprocessing of the pyrosequencing data for the candidates, the data was imported into R. Summary statistics for the CpGs measured in each candidate region were generated along with density plots, this confirmed that the methylation percentage measurements for the CpGs were non-normally distributed and highlighted values that would be classed as outlying. Robust regressions using the *rlm* function in *R* were performed for each CpG at each candidate locus, fitting the following model :

*Methylation ~ batch + childs_age + sex + Bcell + CD4T + CD8T + Eos + Mono + NK + season_of_conception (Gambia only) + intervention*

Where batch is the pyrosequencing batch/plate, and the other variables are as defined in the EWAS methods above. Neu blood cell type was removed to reduce variance inflation. Following this, the *coeftest* function from the *lmtest* package ^22^ was used along with the *vcovHC* function from sandwich ^23^ to perform a Wald test on the heteroscedasticity-consistent estimated coefficients. An FDR cutoff of 5% was specified to account for multiple testing across all the measured sites.

**SM5.2 Region level differential methylation analysis**

Next, to obtain a per candidate, regional estimate of the effect of intervention, an evidence combination method was used taking the p values from the single site robust regression analyses. The *EmpiricalBrownsMethod* package from Bioconductor was used ^24^. This is an empirical adaptation of Brown’s method, which extends Fisher’s p value combination to take into account dependency between statistics, estimating this using the covariance of the measurements themselves (in this case the site level methylation data for the candidates). The regional p-values were then corrected for multiple testing across all candidate regions, again specifying an FDR cutoff of 5 %.

**SM5.3 Season of conception interaction (Gambian only)**

A sensitivity analysis looking at possible interaction with season of conception was performed for the candidates by re running the regressions with the interaction term included:

*Methylation ~ batch + childs_age + sex + Bcell + CD4T + CD8T + Eos + Mono + NK + season_of_conception + intervention + season_of_conception:intervention*

Supplemental Results

**Posthoc Analysis sensitivity analyses**

**Positive control EWAS (Indian cohort only)**

We performed a positive control EWAS in the Indian data using maternal BMI as the predictor variable and child methylation as the outcome. The following model was used: *M ~ ISVs + covariates + intervention group + maternal BMI*. Bacon adjustment was performed to reduce genomic inflation. The results confirmed that an effect size distribution indicative of a non-null effect could be obtained using maternal BMI.

**Maternal BMI interaction analysis (Indian cohort only)**

We did not find evidence for an interaction between maternal BMI and intervention. Similarly, stratified analysis based on the median maternal pre pregnant BMI (19.5) did not indicate that intervention effect sizes differed depending on maternal BMI (Supplemental Figure 3).

**Compliance (Indian cohort only)**

The results showed no significant interaction between intervention group and maternal compliance status on the child’s methylation profile, illustrated by the interaction term. A stratified analysis did not show evidence of effect sizes for intervention being different for the compliance strata (Supplemental Figure 4). Focussing on fully compliant women only (total n=339; 159/180 intervention/control), no CpGs passed FDR <0.05 and no CpGs in the top 10 most significant loci from the main analysis (Table 4) appeared in the top 10 loci in the full compliance analysis (Supplemental Table 16). Although the smaller sample size meant that statistical power was reduced in this analysis, effect sizes (change in methylation Beta values) were again very small, supporting our conclusion from the main analysis that the Indian intervention had no discernible effect on child DNAm.

References

1. Yan, L. *et al.* OSAT: a tool for sample-to-batch allocations in genomics experiments. *BMC Genomics* **13,** 689 (2012).

2. Min, J. L., Hemani, G., Davey Smith, G., Relton, C. & Suderman, M. Meffil: efficient normalization and analysis of very large DNA methylation datasets. *Bioinformatics* (2018). doi:10.1093/bioinformatics/bty476

3. Pidsley, R. *et al.* Critical evaluation of the Illumina MethylationEPIC BeadChip microarray for whole-genome DNA methylation profiling. *Genome Biol.* **17,** 208 (2016).

4. Triche, T. J., Weisenberger, D. J., Van Den Berg, D., Laird, P. W. & Siegmund, K. D. Low-level processing of Illumina Infinium DNA Methylation BeadArrays. *Nucleic Acids Res.* **41,** e90–e90 (2013).

5. Fortin, J.-P. *et al.* Functional normalization of 450k methylation array data improves replication in large cancer studies. *Genome Biol.* **15,** 503 (2014).

6. Guo, Y. *et al.* Illumina human exome genotyping array clustering and quality control. *Nat. Protoc.* **9,** 2643–2662 (2014).

7. Leek, J. T. & Storey, J. D. Capturing Heterogeneity in Gene Expression Studies by Surrogate Variable Analysis. *PLoS Genet.* **3,** e161 (2007).

8. Teschendorff, A. E., Zhuang, J. & Widschwendter, M. Independent surrogate variable analysis to deconvolve confounding factors in large-scale microarray profiling studies. *Bioinformatics* **27,** 1496–1505 (2011).

9. Silver, M. J. *et al.* Independent genomewide screens identify the tumor suppressor VTRNA2-1 as a human epiallele responsive to periconceptional environment. *Genome Biol.* **16,** 118 (2015).

10. van Baak, T. E. *et al.* Epigenetic supersimilarity of monozygotic twin pairs. *Genome Biol.* **19,** 2 (2018).

11. van Iterson, M., van Zwet, E. W. & Heijmans, B. T. Controlling bias and inflation in epigenome- and transcriptome-wide association studies using the empirical null distribution. *Genome Biol.* **18,** 19 (2017).

12. Sharp, G. C. *et al.* Maternal BMI at the start of pregnancy and offspring epigenome-wide DNA methylation: Findings from the pregnancy and childhood epigenetics (PACE) consortium. *Hum. Mol. Genet.* **26,** 4067–4085 (2017).

13. Potdar, R. D. *et al.* Improving women’ s diet quality preconceptionally and during gestation: effects on birth weight and prevalence of low birth weight-a randomized controlled efficacy trial in India (Mumbai Maternal Nutrition Project). *Am J Clin Nutr* **100,** 1257–1268 (2014).

14. Dominguez-Salas, P. *et al.* Maternal nutrition at conception modulates DNA methylation of human metastable epialleles. *Nat. Commun.* **5,** 3746 (2014).

15. Saffari, A. *et al.* Estimation of a significance threshold for epigenome-wide association studies. *Genet. Epidemiol.* **42,** 20–33 (2018).

16. Peters, T. J. *et al.* De novo identification of differentially methylated regions in the human genome. *Epigenetics Chromatin* **8,** 6 (2015).

17. Phipson, B., Maksimovic, J. & Oshlack, A. missMethyl: an R package for analyzing data from Illumina’s HumanMethylation450 platform. *Bioinformatics* **32,** btv560 (2015).

18. Pedersen, B. S., Schwartz, D. A., Yang, I. V. & Kechris, K. J. Comb-p: software for combining, analyzing, grouping and correcting spatially correlated P-values. *Bioinformatics* **28,** 2986–2988 (2012).

19. Pan, H., Holbrook, J. D., Karnani, N. & Kwoh, C. K. Gene, Environment and Methylation (GEM): a tool suite to efficiently navigate large scale epigenome wide association studies and integrate genotype and interaction between genotype and environment. *BMC Bioinformatics* **17,** 299 (2016).

20. Monk, D. *et al.* Recommendations for a nomenclature system for reporting methylation aberrations in imprinted domains. *Epigenetics* **13,** 117–121 (2018).

21. Goeman, J. J. & Bühlmann, P. Analyzing gene expression data in terms of gene sets: Methodological issues. *Bioinformatics* **23,** 980–987 (2007).

22. Zeileis, A. & Hothorn, T. Diagnostic checking in regression relationships. (2002).

23. Zeileis, A. Econometric Computing with HC and HAC Covariance Matrix Estimators. *J. Stat. Softw.* **11,** (2004).

24. Poole, W., Gibbs, D. L., Shmulevich, I., Bernard, B. & Knijnenburg, T. A. Combining dependent P- values with an empirical adaptation of Brown’s method. *Bioinformatics* **32,** i430–i436 (2016).

Supplemental Figures

**Supplemental Figure** **1. CONSORT flowcharts for Indian (top) and Gambian (bottom) cohorts.**This data includes all women and their offspring who participated in the original intervention trials. Text in blue (India) and green (Gambia) highlights children participating in the EMPHASIS follow-up study.

**
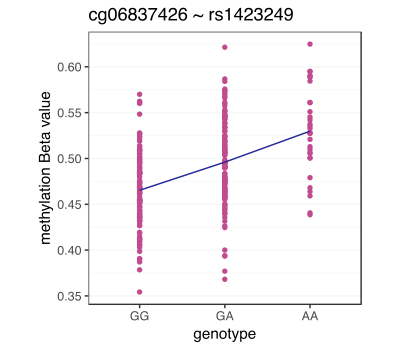
**

**Supplemental Figure** **2. Plot showing the effect of genotype of the mQTL rs1423249 on methylation status at the *ESM1* CpG cg06837426 - the most significant mQTL association identified by the GEM genotype only (G) model.**


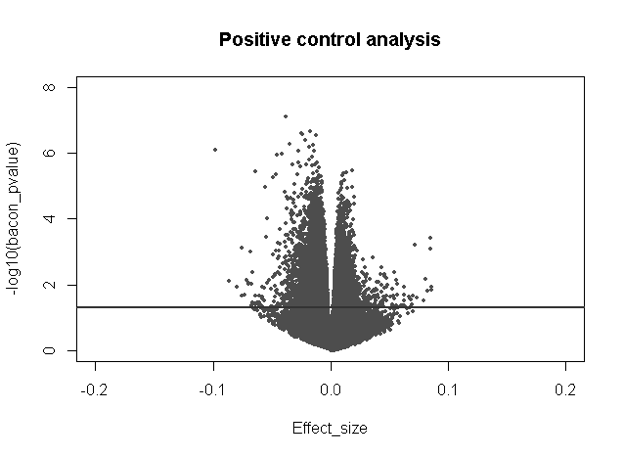


**Supplemental Figure** **3.** **Positive control analysis (Indian EWAS;** n=686**).** Volcano plot showing effect of maternal BMI on child methylation. The horizontal line indicates nominal p=0.05. The x-axis represents the regressional model beta coefficent for maternal BMI and the y-axis shows -log10 p values adjusted for genomic inflation by bacon.


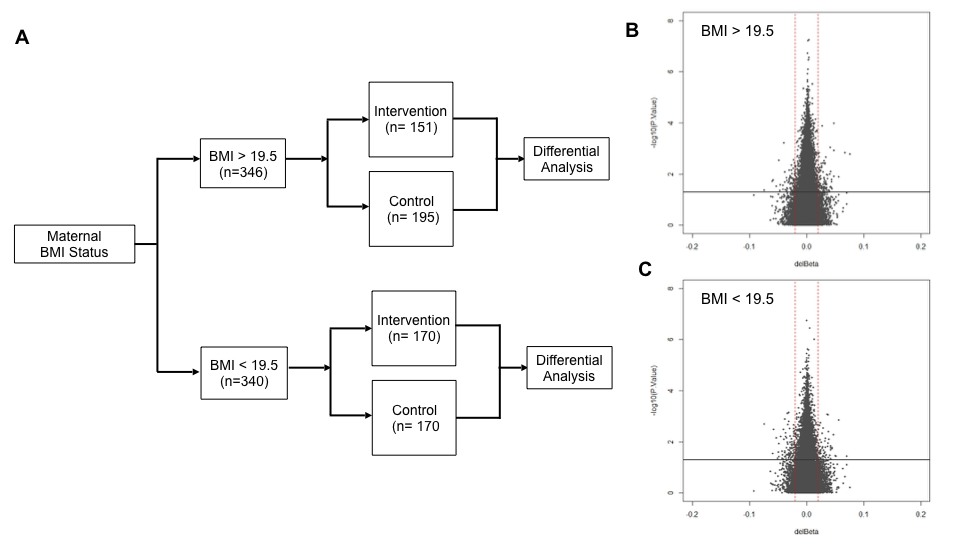


**Supplemental Figure** **4** **Stratified analysis based on median maternal BMI status on child DNA methylation in the Indian cohort**. (A) Flow chart depicting the method of stratification and analysis. Volcano plot illustrating (B) Beta change of DMPs in the BMI>19.5 category and (C) BMI<19.5 category. The dotted vertical red lines indicate delta beta 2% and the horizontal black line represents p value = 0.05.


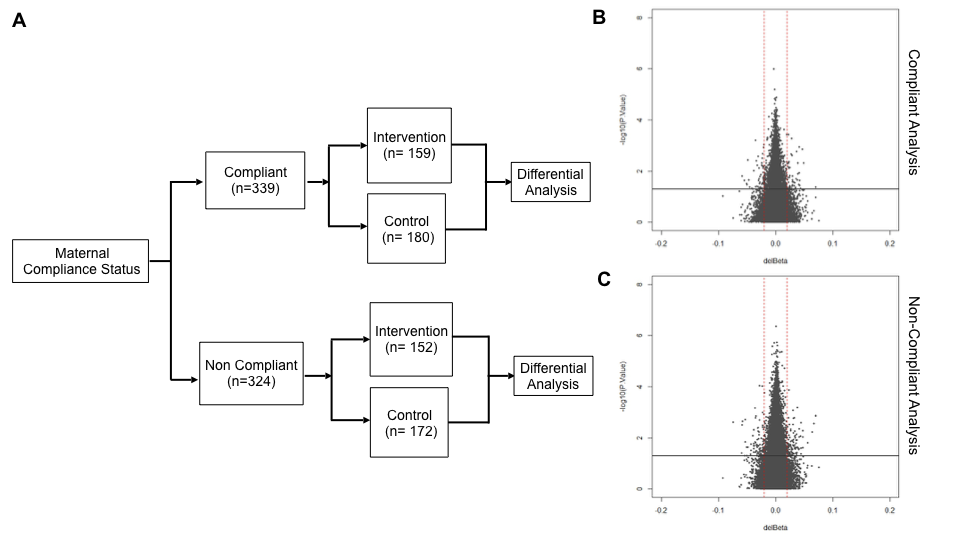


**Supplemental Figure 5.** **Stratified analysis of maternal compliance status on child DNA methylation in the Indian cohort**. (A) Flow chart depicting the method of stratification and analysis. Volcano plots for (B) the compliant and C) non-compliant category. The dotted vertical red lines indicate delta Beta 2% and the horizontal black line represents p value = 0.05.

**A.**


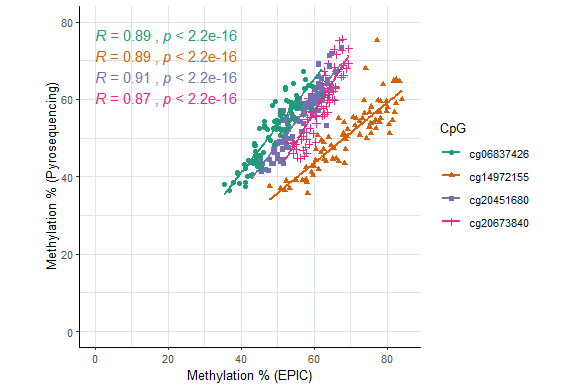


**B.**


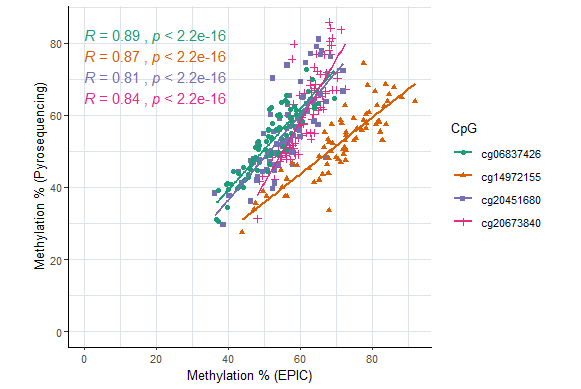


**Supplemental Figure 6. Technical validation of 4 *ESM1* CpGs by pyrosequencing (n=92) showing concordance with EPIC array measurements for (A) Gambian (B) Indian cohort.**

R: Spearman’s rho.

**A.**

**
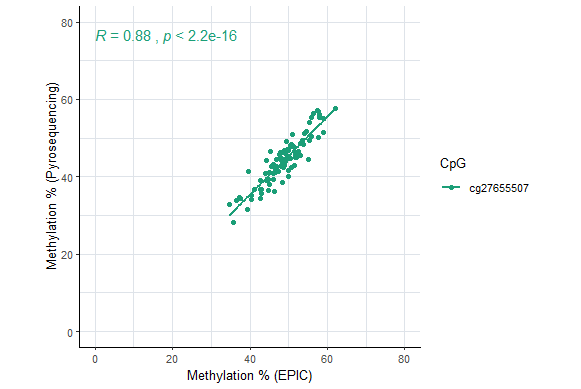
**

**B.**


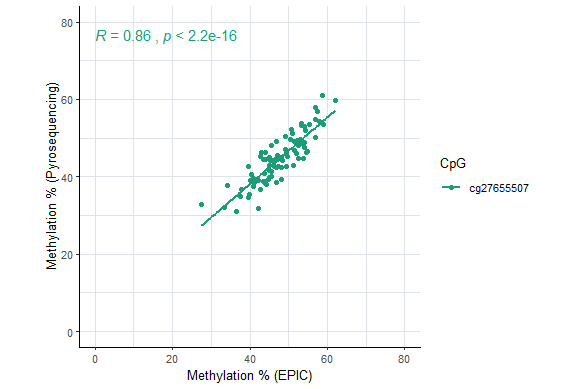


**Supplemental Figure** **7.** **Technical validation of 1 *LZTS1* CpG by pyrosequencing (n=92) showing concordance with EPIC array measurements in the (A) Gambian and (B) Indian cohort.** R: Spearman’s rho.

**A.**

**
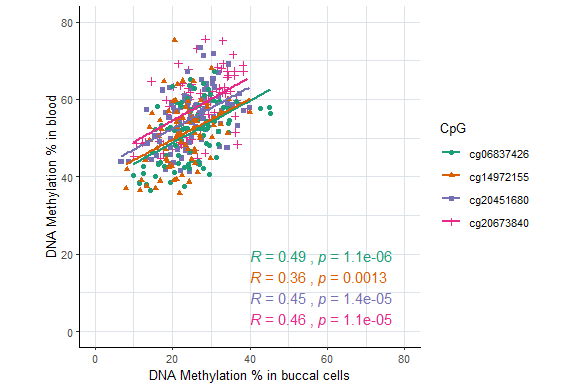
**

**B.**

**
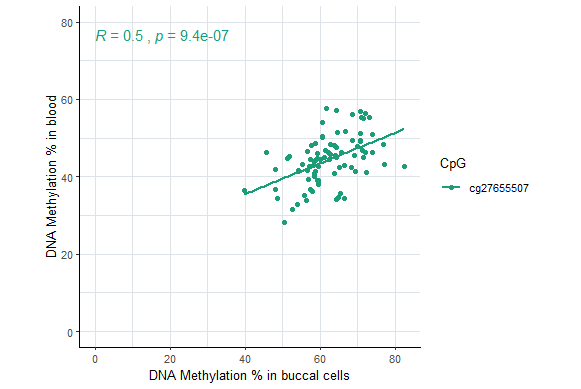
**

**Supplemental Figure 8. Cross tissue comparison showing correlation between percentage methylation values in blood vs buccal cells for (A) *ESM1* and (B) *LZTS1* CpGs.**

R: Spearman’s rho, n= 75-89).

**A.**

**
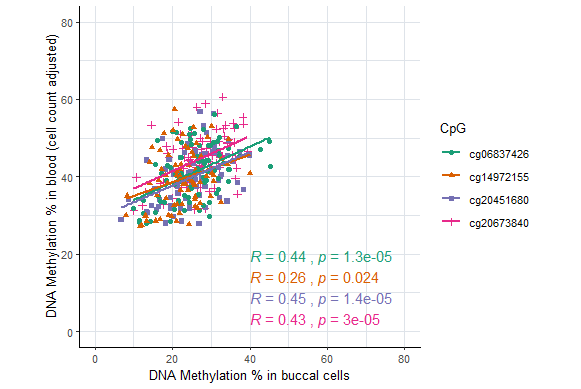
**

**B.**

**
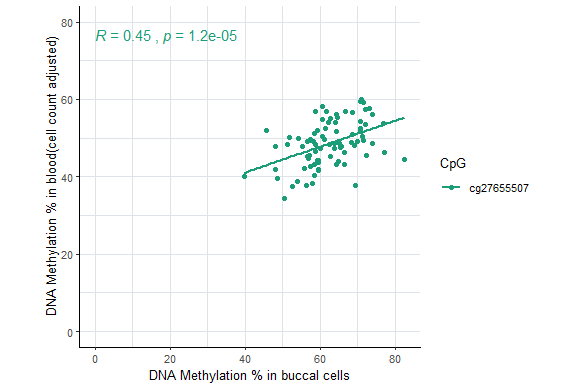
**

**Supplemental Figure 9. Cross tissue comparison showing blood cell composition-adjusted blood vs buccal cell percentage methylation values at *ESM1* (A) and *LZTS1* (B) CpGs.**

R: Spearman’s rho, n=75-89).

**A. Gambian intervention EWAS B. Indian intervention EWAS**


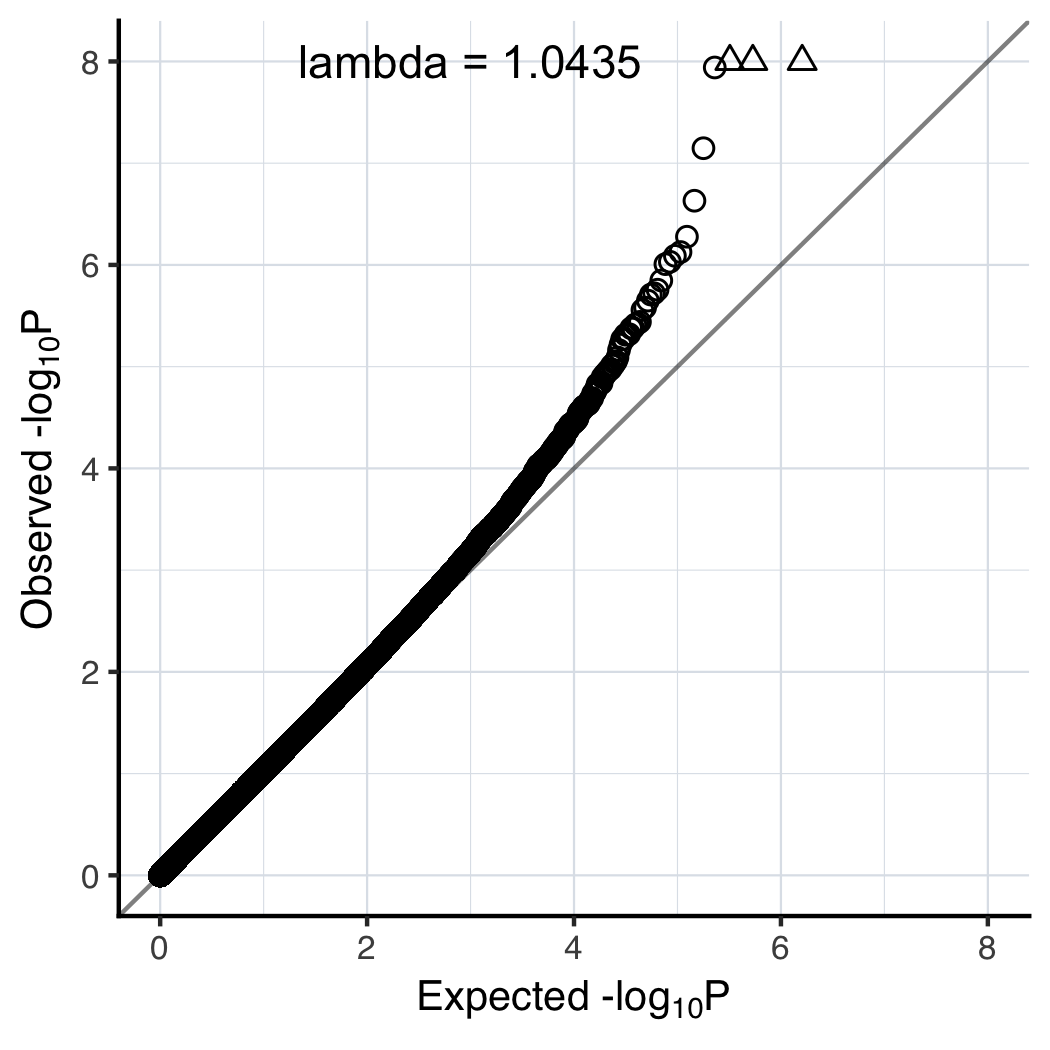

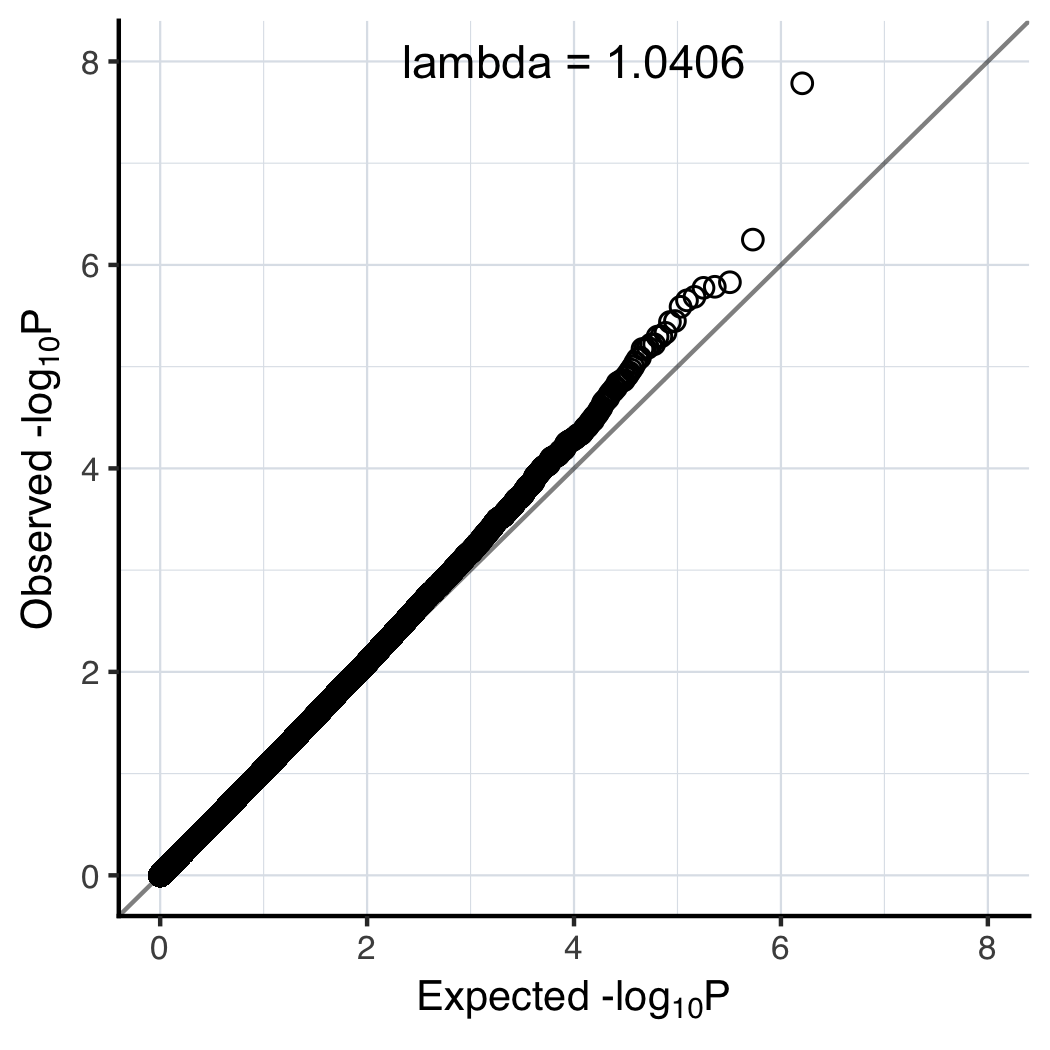


**Supplemental Figure 10.** **Quantile-Quantile (QQ) plots for the (A) Gambian (B) Indian intervention EWAS final models.** A multiple linear regression model was used for EWAS in both Gambian (n=289) and Indian (n=686) cohorts.The triangles indicate a point falling outside the plot area; circles represent all other CpGs analysed.

Supplemental Tables

**Supplemental Table 1: Candidate loci not on the EPIC array assessed by pyrosequencing**

| **Gene** | **ROI coordinates (hg19)** | **Reported associations** | **Ref** |
| --- | --- | --- | --- |
| *H19* | chr11:2024197-2024341 | Maternal preconceptional and pregnancy folic acid intake reduced H19 methylation in infants | 1 |
| *HES1* | chr3:193849141-193849361 | HES1 hypermethylation at birth related with better neurocognitive outcomes and behaviour in children | 2 |
| *IGF2* | chr11:2169457-2169541 | Maternal nutrition before and during pregnancy associated with IGF2 methylation | 3–5 |
| *IGF2* | chr11:2169617-2169751 |  |  |
| *MEG3 (GTL2)* | chr14:101294220-101294391 | Child methylation signature associated with maternal folate levels and birthweight | 6 |
| *NOS3* | chr7:150684570-150684745 | Methylation at NOS3 associated with childhood adiposity and bone composition | 7,8 |
| *PAX8* | chr2:113992866-113993036 | Increased methylation associated with rainy season conception in Gambians | 9 |
| *RBM46* | chr4:155702818-155703110 | Higher DNA methylation reported when conceived in rainy season in The Gambia | 10 |
| *RXRA* | chr9:137215689-137215826 | Methylation inversely associated with maternal carbohydrate intake during pregnancy; Differential methylation linked to childhood adiposity and bone composition | 7,11 |
| *RXRA* | chr9:137215979-137216126 |  |  |

**Supplemental Table 2.** **Primer sequences used for pyrosequencing of the candidate loci**

| **Gene** | **Genomic Location (hg19)** | **Forward Primer** | **Reverse Primer** | **Sequencing Primer** |
| --- | --- | --- | --- | --- |
| *H19* | chr11:2024197-2024341 | TTTGTTGATTTTATTAAGGGAGGT | Btn-CTATAAATAAACCCCAACCAAAC | GTGTGGAATTAGAAGT |
| *HES1* | chr3:193849141-193849361 | AGGGGATAAAGGGGAGTT | Btn-TCACTTCTTTAATCCCCCTATAACACCA | GGTTTGAAAGTAAATAGGT |
| *IGF2* | chr11:2169457-2169541 | GGGGGTTTTAGTAAAAGTTATTGGAT | Btn-ATTTCCCCAAAAAACACAACC | AGTAAAAGTTATTGGATATATAGT |
| *IGF2* | chr11:2169617-2169751 | TGTATGAATGAGTATTTTTAGGGAAATTGT | Btn-CTCCATATCCCCCCTAAATTTAACT | AGGGAAATTGTTTTGG |
| *MEG3 (GTL2)* | chr14:101294220-101294391 | GGGATTTTTGTTTTTTTTTGTAGTAGG | Btn-CCAACCAAAACCCACCTATAAC | TTTGGGGTTGGGGTT |
| *NOS3* | chr7:150684570-150684745 | Btn-GGGAGTTTTTTAGTTGGGGAGTAG | ACAAACCCCTACCACTACCAAAAT | ATCTTAAATTTCCAAATCAC |
| *PAX8* | chr2:113992866-113993036 | GGGGTGGATGAGATTGAGGTTAGA | Btn-CCTTCAATACCTTTCCCCATACTACC | GGTTTGTTTTGAGGAT |
| *RBM46* | chr4:155702818-155703110 | TTGTATGGTGAGGGTTTAG | Btn-TCTAAAACCAAACTACTAAATCT | GTGTTATTTTTTTGATA |
| *RXRA* | chr9:137215689-137215826 | Btn-GGGAAGGTTGAAGGTTTTAGAAG | AACACAAAAACTAAATATAAACCCAAATC | AAACTAAATATAAACCCAAATCT |
| *RXRA* | chr9:137215979-137216126 | Btn-TTTTGGAGTAGGGAGGTTTTATTAG | CAAACACTTCAACCCAACCTTC | AACCTTCCCACCATT |

**Supplemental Table 3.** **Summary statistics for candidate pyrosequenced loci**

|  |  |  | **India** | | | **Gambia** | | |
| --- | --- | --- | --- | --- | --- | --- | --- | --- |
| **Gene** | **CpG** | **Positive Control SD** | **Mean**  **methylation** | **Total N** | **N post QC** | **Mean**  **methylation** | **Total N** | **N post QC** |
| *H19* |  |  |  |  |  |  |  |  |
|  | cg1 | 3.78 | 61.11 | 698 | 667 | 63.36 | 292 | 289 |
|  | cg2 | 1.74 | 57.53 | 698 | 668 | 59.46 | 292 | 289 |
|  | cg3 | 1.87 | 58.05 | 698 | 666 | 60.48 | 292 | 289 |
|  | cg4 | 1.55 | 58.24 | 698 | 663 | 60.53 | 292 | 289 |
| *HES1* |  |  |  |  |  |  |  |  |
|  | cg1 | 2.31 | 48.26 | 698 | 692 | 49.78 | 292 | 291 |
|  | cg2 | 2.07 | 48.04 | 698 | 666 | 49.99 | 292 | 286 |
|  | cg3 | 2.18 | 43.22 | 698 | 662 | 46.76 | 292 | 287 |
|  | cg4 | 2.15 | 55.33 | 698 | 659 | 55.93 | 292 | 284 |
|  | cg5 | 1.86 | 46.78 | 698 | 656 | 48.65 | 292 | 284 |
| *IGF2* |  |  |  |  |  |  |  |  |
|  | cg1 | 1.83 | 47.82 | 698 | 677 | 47.64 | 292 | 290 |
|  | cg2 | 1.78 | 50.43 | 698 | 673 | 49.84 | 292 | 286 |
|  | cg3 | 2.16 | 39.83 | 698 | 674 | 39.34 | 292 | 269 |
|  | cg5 | 1.59 | 57.24 | 698 | 688 | 57.06 | 292 | 291 |
|  | cg6 | 1.20 | 46.19 | 698 | 677 | 47.38 | 292 | 290 |
| *MEG3* |  |  |  |  |  |  |  |  |
|  | cg1 | 1.97 | 54.12 | 698 | 665 | 57.39 | 292 | 290 |
|  | cg2 | 1.42 | 71.60 | 698 | 655 | 75.71 | 292 | 290 |
|  | cg3 | 2.06 | 77.10 | 698 | 656 | 80.46 | 292 | 284 |
|  | cg4 | 1.94 | 74.41 | 698 | 650 | 78.23 | 292 | 277 |
|  | cg5 | 1.67 | 80.25 | 698 | 646 | 82.99 | 292 | 278 |
| *NOS3* |  |  |  |  |  |  |  |  |
|  | cg1 | 3.52 | 67.88 | 698 | 676 | 68.31 | 292 | 291 |
|  |  |  |  |  |  |  |  |  |
| *PAX8* |  |  |  |  |  |  |  |  |
|  | cg1 | 2.42 | 64.38 | 698 | 675 | 66.13 | 292 | 292 |
|  | cg2 | 3.28 | 68.06 | 698 | 684 | 68.90 | 292 | 292 |
|  | cg3 | 2.39 | 63.35 | 698 | 668 | 63.79 | 292 | 292 |
|  | cg4 | 2.59 | 61.64 | 698 | 668 | 62.53 | 292 | 292 |
| *RBM46* |  |  |  |  |  |  |  |  |
|  | cg1 | 3.12 | 67.8 | 698 | 666 | 68.17 | 292 | 291 |
|  | cg2 | 3.74 | 72.13 | 698 | 666 | 72.37 | 292 | 290 |
|  | cg3 | 4.82 | 67.69 | 698 | 662 | 68.22 | 292 | 290 |
|  | cg4 | 5.34 | 59.01 | 698 | 650 | 59.71 | 292 | 289 |
|  | cg5 | 4.06 | 56.09 | 698 | 640 | 57.00 | 292 | 287 |
|  | cg6 | 3.63 | 71.65 | 698 | 634 | 71.89 | 292 | 287 |
|  | cg7 | 4.34 | 62.15 | 698 | 619 | 62.54 | 292 | 282 |
|  | cg8 | 2.08 | 62.65 | 698 | 613 | 64.01 | 292 | 281 |
| *RXRA* |  |  |  |  |  |  |  |  |
|  | cg1 | 1.41 | 81.29 | 698 | 696 | 79.84 | 292 | 291 |
|  | cg2 | 2.02 | 71.79 | 698 | 696 | 71.57 | 292 | 291 |
|  | cg3 | 1.05 | 88.97 | 698 | 688 | 88.08 | 292 | 291 |
|  | cg4 | 1.61 | 73.84 | 698 | 692 | 71.64 | 292 | 291 |
|  | cg5 | 3.60 | 75.13 | 698 | 671 | 76.30 | 292 | 289 |
|  | cg6 | 1.35 | 90.65 | 698 | 676 | 90.80 | 292 | 287 |
|  | cg9 | 1.80 | 80.70 | 698 | 696 | 78.95 | 292 | 290 |
|  | cg10 | 1.43 | 82.02 | 698 | 683 | 81.18 | 292 | 290 |
|  | cg11 | 0.78 | 95.54 | 698 | 670 | 95.54 | 292 | 275 |
|  | cg12 | 0.83 | 89.68 | 698 | 664 | 89.61 | 292 | 275 |

N, number of subjects

**Supplemental Table 4: Lookup of Gambian top loci in Indian EWAS results**

|  |  |  | **GAMBIA** | | | | | **INDIA** | | | | |
| --- | --- | --- | --- | --- | --- | --- | --- | --- | --- | --- | --- | --- |
| **CpG** | **Location** | **Gene** | **p value** | **FDR** | **Delta Beta** | **t** | **Rank** | **p value** | **FDR** | **Delta Beta** | **t** | **Rank** |
| cg20451680 | chr5:54281336 | *ESM1* | *6.36E-10* | *0.0005* | *-0.0331* | *6.4091* | 1 | 0.3097 | 0.9694 | -0.0018 | 0.4720 | 256580 |
| cg14972155 | chr5:54281198 | *ESM1* | *5.10E-09* | *0.0014* | *-0.0498* | *6.0363* | 2 | 0.5052 | 0.9836 | 0.0020 | 0.0884 | 412495 |
| cg20673840 | chr5:54281362 | *ESM1* | *5.39E-09* | *0.0014* | *-0.0246* | *6.0261* | 3 | 0.236 | 0.9587 | -0.0019 | 0.6302 | 197719 |
| cg06837426 | chr5:54281271 | *ESM1* | *1.15E-08* | *0.0023* | *-0.0303* | *5.8868* | 4 | 0.6444 | 0.9894 | -0.0012 | -0.0712 | 523059 |
| cg09612591 | chr2:79519823 | *CTNNA2* | *7.13E-08* | *0.0114* | *-0.0299* | *5.5389* | 5 | 0.5599 | 0.9862 | -0.0034 | 0.3395 | 455881 |
| cg05676441 | chr5:19988800 | *CDH18* | *2.34E-07* | *0.0313* | *-0.0286* | *5.3037* | 6 | 0.0744 | 0.9149 | 0.0010 | 1.3417 | 65277 |
| cg27655507 | chr8:20159789 | *LZTS1* | *5.28E-07* | *0.0605* | *-0.0283* | *5.1378* | 7 | 0.0114 | 0.8184 | -0.0066 | 2.1971 | 11224 |
| cg21180956 | chr5:54281478 | *ESM1* | *7.47E-07* | *0.0717* | *-0.0320* | *5.0660* | 8 | 0.3018 | 0.9684 | -0.0023 | 0.5936 | 250299 |
| cg10631947 | chr5:54281668 | *ESM1* | *9.31E-07* | *0.0717* | *0.0176* | *5.0200* | 10 | 0.6262 | 0.9887 | 0.0013 | 0.0373 | 508691 |
| cg24403549 | chr5:54281572 | *ESM1* | *3.85E-06* | *0.1463* | *-0.0281* | *4.7149* | *21* | 0.079 | 0.9158 | -0.0043 | 1.2966 | 69286 |
| cg15631458 | chr8:20160009 | *LZTS1* | *4.19E-06* | *0.1463* | *-0.0242* | *4.6964* | 22 | 0.0092 | 0.8172 | -0.0062 | 2.1988 | 9011 |
| cg13106512 | chr5:54281507 | *ESM1* | *4.80E-06* | *0.1497* | *-0.0234* | *4.6664* | 24 | 0.1906 | 0.9504 | -0.0024 | 0.8238 | 161095 |
| cg20059697 | chr5:54281687 | *ESM1* | *2.10E-05* | *0.3008* | *-0.0236* | *4.3292* | 56 | 0.2052 | 0.9539 | -0.0016 | 0.7244 | 172796 |
| cg09772075 | chr8:20159446 | *LZTS1* | *6.95E-05* | *0.4160* | *0.0192* | *4.0399* | 134 | 0.1018 | 0.9241 | -0.0032 | 1.2535 | 88509 |
| cg16462183 | chr5:54281733 | *ESM1* | *1.25E-03* | *0.7224* | *0.0191* | *3.2622* | 1375 | 0.2409 | 0.9595 | -0.0016 | 0.7288 | 201653 |
| cg11809339 | chr8:20159382 | *LZTS1* | *6.02E-03* | *0.8402* | *0.0074* | *2.7685* | 5730 | 0.768 | 0.9936 | 0.0008 | -0.0513 | 620787 |

Status of Gambian top hits (nCpGs=16) in Indian EWAS results. *Bacon* inflation-adjusted p values and FDR corrected values are given for the Indian dataset, along with delta Beta, t-statistic and rank (by p value).

**Supplemental Table 5:** **Gambian candidate locus regional analysis**

| **Candidate** | **p value** | **FDR** |
| --- | --- | --- |
| *H19* | 1.22E-02 | 9.75E-02 |
| *PAX8* | 4.65E-02 | 1.86E-01 |
| *NOS3* | 1.48E-01 | 3.39E-01 |
| *RBM46* | 1.70E-01 | 3.39E-01 |
| *HES1* | 3.26E-01 | 4.98E-01 |
| *IGF2* | 3.73E-01 | 4.98E-01 |
| *MEG3* | 7.38E-01 | 8.08E-01 |
| *RXRA* | 8.08E-01 | 8.08E-01 |

Nominal p values are calculated by combination of individual site level p values (see Supplemental Methods SM5.2). FDR accounts for multiple testing of candidate regions.

**Supplemental Table 6:** **Indian candidate locus region level analysis**

| **Candidate** | **p value** | **FDR** |
| --- | --- | --- |
| *RBM46* | 0.1456 | 0.8559 |
| *MEG3* | 0.2342 | 0.8559 |
| *PAX8* | 0.5577 | 0.8559 |
| *RXRA* | 0.6322 | 0.8559 |
| *NOS3* | 0.6366 | 0.8559 |
| *HES1* | 0.6972 | 0.8559 |
| *IGF2* | 0.8397 | 0.8559 |
| *H19* | 0.8559 | 0.8559 |

The p values are calculated by combination of individual site level p values (see Supplemental Methods SM5.2). FDR accounts for multiple testing across all candidate regions.

**Supplemental Table 7:** **Gambian candidate locus single site analysis**

| **Candidate** | **CpG** | **Coef.** | **Std. Error** | **Statistic** | **p value** | **FDR** |
| --- | --- | --- | --- | --- | --- | --- |
| ***H19*** | 1 | -1.0999 | 0.4631 | -2.3752 | 1.75E-02 | 2.46E-01 |
| *n = 283* | 2 | -0.5553 | 0.3419 | -1.6242 | 1.04E-01 | 4.33E-01 |
|  | 3 | -1.0824 | 0.3870 | -2.7970 | 5.16E-03 | 1.93E-01 |
|  | 4 | -0.9950 | 0.3821 | -2.6044 | 9.20E-03 | 1.93E-01 |
| ***PAX8*** | 1 | 3.9753 | 2.1462 | 1.8522 | 6.40E-02 | 3.56E-01 |
| *n = 286* | 2 | 4.2733 | 2.1552 | 1.9828 | 4.74E-02 | 3.33E-01 |
|  | 3 | 4.1097 | 2.0256 | 2.0289 | 4.25E-02 | 3.33E-01 |
|  | 4 | 3.9152 | 1.9756 | 1.9818 | 4.75E-02 | 3.33E-01 |

The results are shown for the two regions *H19* and *PAX8* which both contained multiple sites reaching nominal significance (p value<0.05). FDR accounts for multiple testing for all CpGs tested across all candidates. coef.- beta coefficient, n - number of samples.

**Supplemental Table 8:** **Significant mQTL (FDR<5%) for Gambian intervention-associated DMPs and CpGs within DMRs as identified by GEM using a genotype-only (G) model**

| **CpG** | **CpG location** | **Gene** | **SNP** | **SNP location** | **Coef.** | **p value** | **FDR** |
| --- | --- | --- | --- | --- | --- | --- | --- |
| cg06837426 | chr5:54281271 | *ESM1* | rs1423249 | chr5:54272446 | 0.0302 | 3.32E-13 | 1.13E-06 |
| cg20673840 | chr5:54281362 | *ESM1* | rs1423249 | chr5:54272446 | 0.0232 | 5.62E-13 | 1.13E-06 |
| cg20451680 | chr5:54281336 | *ESM1* | rs1423249 | chr5:54272446 | 0.0286 | 3.00E-12 | 4.01E-06 |
| cg14972155 | chr5:54281198 | *ESM1* | rs1423249 | chr5:54272446 | 0.0449 | 2.86E-11 | 2.87E-05 |
| cg20059697 | chr5:54281687 | *ESM1* | rs1423249 | chr5:54272446 | 0.0266 | 1.59E-09 | 1.28E-03 |
| cg13106512 | chr5:54281507 | *ESM1* | rs1423249 | chr5:54272446 | 0.0233 | 2.58E-09 | 1.73E-03 |
| cg21180956 | chr5:54281478 | *ESM1* | rs1423249 | chr5:54272446 | 0.0276 | 2.12E-08 | 1.21E-02 |

A total of seven CpGs in *ESM1* were identified as being influenced by the same *cis* mQTL: rs1423249. The beta coefficient value (coef.) is the additive effect of each variant allele on methylation status of the CpG, which is given along with the p value and FDR adjusted p value. mQTL, methylation quantitative trait loci; GEM, Gene Environment and Methylation package in R; DMPs, differentially methylated positions; DMRs, differentially methylated regions; SNP, single nucleotide polymorphism; FDR, false discovery rate.

**Supplemental Table 9.** **Further modelling of *ESM1* CpGs associated with the mQTL rs1423249**

|  | **adj.R squared** | | | | **AIC** | | | |
| --- | --- | --- | --- | --- | --- | --- | --- | --- |
| **CpG** | **G** | **E** | **G + E** | **G x E** | **G** | **E** | **G + E** | **G x E** |
| cg06837426 | 0.2500 | 0.1888 | 0.3134 | 0.3109 | -984.06 | -972.46 | -1008.42 | -1006.45 |
| cg20673840 | 0.2797 | 0.2327 | 0.3411 | 0.3389 | -1125.04 | -1121.38 | -1149.56 | -1147.68 |
| cg20451680 | 0.3101 | 0.2828 | 0.3802 | 0.3798 | -987.51 | -988.18 | -1017.22 | -1016.10 |
| cg14972155 | 0.2214 | 0.1967 | 0.2967 | 0.2955 | -700.98 | -700.38 | -729.17 | -727.73 |
| cg20059697 | 0.3476 | 0.2997 | 0.3724 | 0.3700 | -939.44 | -931.11 | -949.59 | -947.59 |
| cg13106512 | 0.4196 | 0.3890 | 0.4465 | 0.4449 | -1004.84 | -1002.26 | -1017.47 | -1015.68 |
| cg21180956 | 0.3459 | 0.3307 | 0.3846 | 0.3823 | -871.85 | -876.94 | -888.33 | -886.33 |

For all CpGs, the G + E model that includes both genotype and the intervention as main effects (and including the same adjustment covariates as the main EWAS analysis) provides the best model fit (largest adjusted R^2^ and smallest AIC scores). G model: genotype + covariates; E: intervention + covariates; G + E: genotype + intervention + covariates; G x E: genotype + intervention + genotype x intervention + covariates. mQTL, methylation quantitative trait loci; AIC, Akaike Information Criterion

**Supplemental Table 10. Estimated coefficients and p values for the best fitting G + E model for *ESM1* CpGs associated with the mQTL rs1423249**

|  | **rs1423249** | | **Intervention** | |
| --- | --- | --- | --- | --- |
| **CpG** | **Coef.** | **p value** | **Coef.** | **p value** |
| cg06837426 | 0.0263 | 6.10E-11 | -0.0249 | 7.19E-07 |
| cg20673840 | 0.0201 | 1.40E-10 | -0.0195 | 6.65E-07 |
| cg20451680 | 0.0243 | 7.11E-10 | -0.0270 | 5.47E-08 |
| cg14972155 | 0.0379 | 5.25E-09 | -0.0436 | 1.13E-07 |
| cg20059697 | 0.0236 | 7.80E-08 | -0.0185 | 7.67E-04 |
| cg13106512 | 0.0204 | 1.57E-07 | -0.0181 | 2.22E-04 |
| cg21180956 | 0.0235 | 1.43E-06 | -0.0256 | 3.32E-05 |

For all CpGs, the intervention and allelic main effects are similar in magnitude, and both are highly significant. mQTL, methylation quantitative trait loci; coef., beta coefficient value.

**Supplemental Table 11. PhenoScanner results using the *ESM1* mQTL rs1423249 as the query SNP, along with other SNPs in LD (r^2^ < 0.8)**

| **Query SNP** | **Query coords** | **LD SNP** | **LD SNP coords** | **r^2^ (query-LD SNP)** | **Trait** | **n** | **p** |
| --- | --- | --- | --- | --- | --- | --- | --- |
|  |  | rs1023675 | chr5:54265606 | 0.9905 | Self-reported pericarditis | 337159 | 9.37E-07 |
|  |  | rs12522821 | chr5:54266360 | 0.9905 | Self-reported pericarditis | 337159 | 9.63E-07 |
|  |  | rs6898099 | chr5:54266917 | 0.9813 | Self-reported pericarditis | 337159 | 1.08E-06 |
|  |  | rs1423249 | chr5:54272446 | 1 | Self-reported pericarditis | 337159 | 1.51E-06 |
| rs1423249 | chr5:54272446 | rs1023673 | chr5:54265411 | 0.9721 | Self-reported pericarditis | 337159 | 1.66E-06 |
|  |  | rs6898099 | chr5:54266917 | 0.9813 | Acute renal failure | 337199 | 3.63E-05 |
|  |  | rs1023675 | chr5:54265606 | 0.9905 | Acute renal failure | 337199 | 3.70E-05 |
|  |  | rs12522821 | chr5:54266360 | 0.9905 | Acute renal failure | 337199 | 3.80E-05 |
|  |  | rs1423249 | chr5:54272446 | 1 | Acute renal failure | 337199 | 3.83E-05 |
|  |  | rs1023673 | chr5:54265411 | 0.9721 | Acute renal failure | 337199 | 5.65E-05 |

The remaining columns provide information on the identified trait associations including details on the study, population, and summary statistics. Associations with p values < 1E-04 are reported here. A Bonferroni-adjusted p value threshold, accounting for the 2,419 traits tested at alpha=0.05, is p_Bonf = 2.07 x 10^-5^. All reported associations are from the UKBB GWAS of ~337,000 individuals of European descent ^12^. LD, linkage disequilibrium; mQTL, methylation quantitative trait loci.; coords, coordinates; n=number of subjects.

**Supplemental Table 12.** **Comparison of Gambian EWAS DMPs (FDR < 5%) obtained using different modelling strategies showing consistency of coefficient estimates and ranks for the identified DMPs**

|  | **Model** | **PCs** | | **All** | | **All no BCCs** | | **SVs** | |
| --- | --- | --- | --- | --- | --- | --- | --- | --- | --- |
| **CpG** | **Gene** | **Coef.** | **Rank** | **Coef.** | **Rank** | **Coef.** | **Rank** | **Coef.** | **Rank** |
| cg20451680 | *ESM1* | -0.1909 | 1 | -0.1670 | 3 | -0.1825 | 1 | -0.1929 | 1 |
| cg14972155 | *ESM1* | -0.3549 | 2 | -0.3252 | 4 | -0.3406 | 3 | -0.3503 | 3 |
| cg20673840 | *ESM1* | -0.1477 | 3 | -0.1310 | 5 | -0.1410 | 6 | -0.1483 | 2 |
| cg06837426 | *ESM1* | -0.1773 | 4 | -0.1562 | 8 | -0.1674 | 4 | -0.1732 | 4 |
| cg09612591 | *CTNNA2* | -0.1875 | 5 | -0.1940 | 1 | -0.1971 | 2 | -0.1883 | 5 |
| cg05676441 | *CDH18* | -0.1726 | 6 | -0.1718 | 9 | -0.1836 | 5 | -0.1710 | 7 |

Ranks are by p value amongst all 803,120 CpGs tested. PCs – principal components (primary) model, ALL – model including known technical and biological variables and estimated blood cell counts, ALL no BCCs –ALL model without blood cell counts, SVs – surrogate variables model; coef.- beta coefficient, EWAS, epigenome-wide association analysis; FDR, false discovery rate; DMPs, differentially methylated positions.

**Supplemental Table 13.** **Sex sensitivity analysis assessing potential influence of uneven distribution of sexes between intervention and control groups at the 6 Gambian DMPs**

| **CpG** | **Gene** | **Rank** | **Coef.** | **95% CI lower** | **95% CI upper** |
| --- | --- | --- | --- | --- | --- |
| cg20451680 | *ESM1* | 1 | -0.1909 | -0.1605 | -0.2140 |
| cg14972155 | *ESM1* | 2 | -0.3549 | -0.2950 | -0.4045 |
| cg20673840 | *ESM1* | 3 | -0.1477 | -0.1218 | -0.1661 |
| cg06837426 | *ESM1* | 4 | -0.1773 | -0.1429 | -0.2002 |
| cg09612591 | *CTNNA2* | 5 | -0.1875 | -0.1527 | -0.2174 |
| cg05676441 | *CDH18* | 6 | -0.1726 | -0.1335 | -0.1977 |

A resampling based approach was used to repeatedly simulate smaller balanced subsamples (n=232, M:F = 1:1). Results from regression models including observed rank and model coefficients reported in the main EWAS findings are shown for reference. Simulation results were used to obtain lower and upper 95% confidence intervals (95% Cis) for the model coefficient for intervention. All coefficient estimates fall within the 95% CIs, indicating reported associations are not significantly influenced by the sex imbalance. coef.- beta coefficient, DMPs, differentially methylated positions.

**Supplemental Table 14.** **Sensitivity analysis to assess potential interaction between intervention and Gambian season of conception for EWAS CpGs at FDR<5%**

| **CpG** | **Gene** | **Coef.** | **p value** | **FDR** |
| --- | --- | --- | --- | --- |
| cg20451680 | *ESM1* | 0.0755 | 0.2522 | 0.7566 |
| cg14972155 | *ESM1* | 0.1581 | 0.2208 | 0.7566 |
| cg20673840 | *ESM1* | 0.0598 | 0.2723 | 0.7566 |
| cg06837426 | *ESM1* | 0.0508 | 0.4458 | 0.7997 |
| cg09612591 | *CTNNA2* | 0.0319 | 0.6702 | 0.9287 |
| cg05676441 | *CDH18* | -0.0078 | 0.9134 | 0.9287 |

Regression models using the primary EWAS model with an added SoC x intervention interaction term indicate that no CpGs show evidence for an interaction with seasonality. The statistics given are for the interaction term. coef.- beta coefficient, EWAS, epigenome-wide association analysis; FDR, false discovery rate; SoC, season of conception.

**Supplemental Table 15.** **Test for interaction between intervention and Gambian season of conception for candidate genes in the Gambian cohort**

|  |  | *SoC interaction model* | | | |
| --- | --- | --- | --- | --- | --- |
|  | **Term:** | **1. intervention** | | **2. intervention : SoC** | |
| **Candidate** |  | **p** | **FDR** | **p** | **FDR** |
| *RBM46* | | 2.32E-02 | 1.45E-01 | 6.94E-02 | 4.28E-01 |
| *PAX8* | | 3.62E-02 | 1.45E-01 | 4.10E-01 | 7.99E-01 |
| *H19* | | 5.82E-02 | 1.55E-01 | 7.11E-01 | 7.99E-01 |
| *IGF2* | | 1.44E-01 | 2.82E-01 | 1.07E-01 | 4.28E-01 |
| *NOS3* | | 1.76E-01 | 2.82E-01 | 6.31E-01 | 7.99E-01 |
| *MEG3* | | 6.65E-01 | 7.68E-01 | 7.60E-01 | 7.99E-01 |
| *HES1* | | 6.72E-01 | 7.68E-01 | 3.78E-01 | 7.99E-01 |
| *RXRA* | | 9.35E-01 | 9.35E-01 | 7.99E-01 | 7.99E-01 |

Region-level nominal p and FDR adjusted p values are shown. These were obtained by re-running the primary candidates regression models and including the interaction. A regional level signal was calculated based on 1. the intervention term and 2. the SoC-intervention interaction term. No regions show evidence for an interaction. FDR, false discovery rate; SoC, season of conception.

**Supplemental Table 16.** **Indian cohort: Top ten most significant associations from intervention EWAS in compliant individuals only (n=339)**

| **CpG** | **Chromosome** | **Location** | **Gene** | **Delta Beta** | **P value** | **FDR** |
| --- | --- | --- | --- | --- | --- | --- |
| cg15457883 | chr10 | 52385225 | *SGMS1* | 0.001906 | 1.65E-07 | 0.132345 |
| cg06001803 | chr19 | 39868139 | *SAMD4B* | 0.001112 | 5.07E-07 | 0.132345 |
| cg01453692 | chr2 | 98264214 | *COX5B* | 0.000883 | 5.76E-07 | 0.132345 |
| cg13607699 | chr17 | 42295918 | *UBTF* | 0.006273 | 6.59E-07 | 0.132345 |
| cg21682382 | chr1 | 32538062 | *TMEM39B* | 0.001226 | 8.96E-07 | 0.143925 |
| og26931862 | chr12 | 54349169 | *HOXC12* | 0.002614 | 1.51E-06 | 0.183329 |
| cg03709178 | chr17 | 46621057 | *HOXB-AS1; HOXB2* | 0.000829 | 1.60E-06 | 0.183329 |
| cg11477110 | chr19 | 3985654 | *EEF2* | 0.001832 | 3.33E-06 | 0.313036 |
| cg14043411 | chr14 | 105219857 | *SIVA1* | 0.000291 | 3.66E-06 | 0.313036 |
| cg12352820 | chr6 | 13616043 | *NOL7* | 0.001589 | 4.20E-06 | 0.313036 |

Given for each probe are the Illumina probe ID, chromosome and genomic position (hg19), annotated gene (EPIC manifest), change in mean Beta between intervention and control groups, regression p value for the effect of intervention group, and the FDR adjusted p vaiue.

**Supplemental Table 17.** **Primers for Site- and Region-Specific Methylation Analyses used for Technical Validation**

| **Gene** | **Primer** | **Sequence** | **CpG ID** | **Coordinate** |
| --- | --- | --- | --- | --- |
| *LZTS1* | Forward | TGTATAGAAGGGAAAGGTGTTTGTGTTTAA | cg27655507 | Chr8: 20159789 |
|  | Sequencing | GGTAGAGAGTATGTAGGTT |  |  |
|  | Reverse | CTATAATAAACAAAAAAAACCTCCAAA- Btn | |  |
| *ESM1* | Forward | AGTTTGGAYGGTTGTTTTTTATTAGTAAAG | cg14972155,cg06837426, cg20451680,cg20673840 | Chr5: 54281198-54281362 |
|  | Sequence 2 | GGTGGTYGTTTGGAGTAA |  |  |
|  | Sequence 3 | GTGATAGTAGTGAGTGTAAA |  |  |
|  | Sequence 1 | AAAGATTAAGATTGGAGAG |  |  |
|  | Reverse | CACTTCATACCATCCATACCTAAAACTATA-Btn |  |  |

**Supplemental Table 18. Comparison of methylation level at *ESM1* and *LZTS1* CpG and Spearman Correlation coefficient for whole blood and Buccal swab comparisons**

Unadjusted and adjusted for blood cell count. Wb, whole blood; Buc, buccal swab; Spearman rho, correlation coefficient.

|  |  |  |  |  | **Unadjusted** | | **Adjusted - blood cell count** | |
| --- | --- | --- | --- | --- | --- | --- | --- | --- |
| **CpG** | **Gene** | **Wb meth%** | **Buc meth%** | **N** | **Spearman rho** | **P value** | **Spearman rho** | **P value** |
| cg14972155 | *ESM1* | 51.08 | 21.89 | 75 | 0.3637 | 0.001339 | 0.2606 | 0.02391 |
| cg06837426 |  | 51.75 | 25.04 | 89 | 0.4895 | 1.13E-06 | 0.4445 | 1.28E-05 |
| cg20451680 |  | 55.34 | 24.88 | 86 | 0.4497 | 1.40E-05 | 0.4491 | 1.44E-05 |
| cg20673840 |  | 58.72 | 26.85 | 86 | 0.455 | 1.07E-05 | 0.4336 | 3.04E-05 |
| cg27655507 | *LZTS1* | 44.27 | 62.33 | 85 | 0.5028 | 9.41E-07 | 0.4547 | 1.24E-05 |

References - Supplemental Tables

1. Hoyo, C. *et al.* Methylation variation at IGF2 differentially methylated regions and maternal folic acid use before and during pregnancy. *Epigenetics* **6,** 928–936 (2011).

2. Lillycrop, K. A. *et al.* Association between perinatal methylation of the neuronal differentiation regulator HES1 and later childhood neurocognitive function and behaviour. *Int. J. Epidemiol.* (2015). doi:10.1093/ije/dyv052

3. Cooper, W. N. *et al.* DNA methylation profiling at imprinted loci after periconceptional micronutrient supplementation in humans: results of a pilot randomized controlled trial. *FASEB J.* **26,** 1782–90 (2012).

4. Steegers-Theunissen, R. P. *et al.* Periconceptional maternal folic acid use of 400 μg per day is related to increased methylation of the IGF2 gene in the very young child. *PLoS One* **4,** e7845 (2009).

5. Heijmans, B. T. *et al.* Persistent epigenetic differences associated with prenatal exposure to famine in humans. *Proc. Natl. Acad. Sci. U. S. A.* **105,** 17046–9 (2008).

6. Hoyo, C. *et al.* Erythrocyte folate concentrations, CpG methylation at genomically imprinted domains, and birth weight in a multiethnic newborn cohort. *Epigenetics* **9,** 1120–1130 (2014).

7. Godfrey, K. M. *et al.* Epigenetic Gene Promoter Methylation at Birth Is Associated With Child’s Later Adiposity. *Diabetes* **60,** 1528–1534 (2011).

8. Harvey, N. C. *et al.* Evaluation of methylation status of the eNOS promoter at birth in relation to childhood bone mineral content. *Calcif. Tissue Int.* **90,** 120–7 (2012).

9. Waterland, R. A. *et al.* Season of conception in rural gambia affects DNA methylation at putative human metastable epialleles. *PLoS Genet.* **6,** 1–10 (2010).

10. Dominguez-Salas, P. *et al.* Maternal nutrition at conception modulates DNA methylation of human metastable epialleles. *Nat. Commun.* **5,** 3746 (2014).

11. Harvey, N. C. *et al.* Childhood bone mineral content is associated with methylation status of the RXRA promoter at birth. *J. Bone Miner. Res.* **29,** 600–7 (2014).

12. Sudlow, C. *et al.* UK Biobank: An Open Access Resource for Identifying the Causes of a Wide Range of Complex Diseases of Middle and Old Age. *PLoS Med.* (2015). doi:10.1371/journal.pmed.1001779
